# Exploring methods for creating or adapting knowledge mobilization products for culturally and linguistically diverse audiences: a scoping review

**DOI:** 10.1186/s13690-024-01334-0

**Published:** 2024-07-22

**Authors:** Sarah A. Elliott, Liza Bialy, Shannon D. Scott, Lisa Hartling

**Affiliations:** 1 Alberta Research Centre for Health Evidence (ARCHE), Faculty of Medicine and Dentistry, Edmonton Clinic Health Academy, University of Alberta, 11405 87 Avenue, Edmonton, AB T6G 1C9 Canada; 2 Faculty of Nursing, Edmonton Clinic Health Academy, University of Alberta, 11405 87 Avenue, Edmonton, AB T6G 1C9 Canada

**Keywords:** Language, Stakeholder participation, Knowledge mobilization, Adaptation, Pamphlets, Mobile applications

## Abstract

**Background:**

Connecting end-users to research evidence has the power to improve patient knowledge and inform health decision-making. Differences in the culture and language of the end users may shape the effectiveness of knowledge mobilization (KMb). This scoping review set out to understand current approaches and methods when creating or adapting KMb products for culturally and linguistically diverse (CALD) audiences.

**Methods:**

We searched 3 databases (Ovid Medline, CINAHL via EBSCOhost, PsychINFO) from 2011 until August 2023. We included any literature about KMb product creation or adaptation processes serving CALD communities. A primary reviewer screened all identified publications and a second reviewer screened publications excluded by the primary. Data were extracted using a standardized form by one reviewer and verified by a second reviewer. Studies were categorized by type of adaptations (‘surface’ and/or ‘deep’ structure) and mapped based on type of stakeholder engagement used in the research approach (i2S model), and end-user involvement (content, design, evaluation and dissemination) in KMb product creation or adaptation.

**Results:**

Ten thousand two hundred ninety-nine unique titles and abstracts were reviewed, 670 full-text studies were retrieved and reviewed, and 78 studies were included in final data extraction and mapping. Twenty-four studies (31%) created or adapted exclusively text-based KMb products such as leaflets and pamphlets and 49 (63%) produced digital products such as videos (*n* = 16, 33%), mobile applications (*n* = 14, 29%), and eHealth websites (*n* = 7, 14%). Twenty-five studies (32%) reported following a framework or theory for their creation or adaptation efforts. Twenty-eight studies (36%) engaged stakeholders in the research approach. Nearly all (96%) involved end-users in creating or adapting the KMb products through involvement in content development (*n* = 64), design features (*n* = 52), evaluation (*n* = 44) and dissemination (*n* = 20). Thirty-two (41%) studies included reflections from the research teams on the processes for creating or adapting KMb products for CALD communities.

**Conclusion:**

Included studies cited a variety of methods to create or adapt KMb products for CALD communities. Successful uptake of created or adapted KMb products was often the result of collaboration and involvement with end-users for more applicable, accessible and meaningful products. Further research developing guidance and best practices is needed to support the creation or adaptation of KMb products with CALD communities.

**Registration:**

Protocol submitted to Open Science Framework on August 16, 2022 (https://osf.io/9jcw4/).

**Supplementary Information:**

The online version contains supplementary material available at 10.1186/s13690-024-01334-0.

**Table:** 

**Text box 1. Contributions to the literature**
• Adaptations found as a part of this scoping review show a wide range of processes used and reveal gaps in what current approaches suggest to be effective.
• Although many of the studies we reviewed claimed to culturally adapt health-related information, they were excluded since they only performed linguistic level translations.
• Theoretical frameworks or evidence-based best practices need to be developed to guide cultural adaptation of health-related information for all levels of consumers (e.g., parents, adolescents, etc.).

## Background

Knowledge mobilization (KMb) (an umbrella term encapsulating knowledge translation, knowledge transfer, and knowledge exchange) [1] involves synthesis, dissemination, transfer and exchange to ensure evidence is accessible, understandable and useful to knowledge users [2, 3]. KMb encompasses a variety of activities, including dissemination of research evidence to increase knowledge users’ access to research, as well as efforts to build and maintain relationships with knowledge users to support the uptake of information [4, 5]. Knowledge creation, in the form of KMb products, is one activity that supports the uptake and use of evidence to inform decision making [6].

Within healthcare, KMb products (which present evidence in clear, concise and user-friendly formats) can help patients and families by informing their health-related behaviours and healthcare decisions to improve health outcomes and reduce health system costs [7]. Successful uptake of evidence is contingent on relevance of the KMb products for the target end-user [2]. However, recognized barriers or determinants of effective KMb are differences in culture and language among the end-users of the evidence [8].

While KMb efforts have advanced substantially in the field of health promotion over the last decade, predominant cultures often comprise the accessible pool of engaged end-users [9, 10]. Subsequently, KMb products frequently entail English communication, and mainstream images not conveying relevance to minority cultures. Similarly, most health related KMb products assume end-users possess a certain level of health literacy and are relatively familiar with their healthcare system, which may not represent the experiences of many newcomer cultural groups. Public health agencies (e.g. Health Canada) sometimes provide linguistic translations of healthcare information for common languages; however, exclusive linguistic translation does not guarantee accessibility and relevance of healthcare information for the target communities. Instead, nuanced visuals, relevant terms, and overall cultural sensitivity have proved more desirable for end-users [11].

Resnicow and colleagues [12] have proposed that cultural adaptation consists of two dimensions: surface structure and deep structure. Aspects of culture that are easily observable to external onlookers like language, clothing, and ethnicity would fall under surface structure, while historical and psychological influences on health decisions would fall under deep structure. Though admittedly gray in nature, the delineation of surface and deep aspects provides potential broad categories of cultural adaptation. In terms of the application of cultural sensitivity, efforts to create or adapt KMb products could include surface structures of language and appearance of end-users, as well as deep structures of historical barriers and psychological stressors for end-users [13]. Resnicow and colleagues suggest that both surface and deep structures of cultural knowledge are essential for well-rounded cultural adaptations and encourage the involvement of end-users to understand the nuanced aspects.

Types and extent of stakeholder engagement can also vary in KMb product creation and adaptation [9, 14, 15]. Bammer [16] proposed a modified version of the International Association for Public Participation (IAP2) stakeholder engagement model, where researchers are positioned as support for the directions chosen by stakeholders and end-users (e.g. those who use the resources), rather than decision-makers and researchers themselves. The positioning of end-users as experts in their own information needs and preferences mirrors other public participation approaches often employed by heath researchers (e.g. Participatory Action Research (PAR) [17], Community-Based Participatory Research (CBPR) [18], etc.). Both cultural adaptation and stakeholder and end-user engagement appear to be core pillars in KMb product creation.

While there are several processes (e.g. translation and cultural brokerage, ecological validity model) of cultural adaptation that have been previously applied to adapt health intervention programs [19], and patient reported outcome scales [20], no guidance currently exists on how best to create or adapt KMb products that reach diverse end-user needs. As an initial step towards understanding best practices for effective KMb product creation or adaptation, within healthcare, this scoping review (ScR) aimed to map what approaches researchers have used to create or adapt culturally relevant health related KMb products. The following questions guided this ScR:

1. What *approaches and methods* have researchers used when creating or adapting health related KMb products for culturally and linguistically diverse (CALD) end-users?
2. What are the *key consideratio*ns when creating or adapting KMb products for CALD end-users?

Understanding what methods have previously been used, resources required, as well as key considerations for how best to create or adapt KMb products will support health researchers and healthcare organizations in creating or adapting effective resources for CALD communities.

## Methods

### Review methods

This ScR followed the methodological framework proposed by Arksey and O’Malley [21], enhanced by Levac et al. [22] Specifically, we followed these five steps: (1) identifying the research question(s); (2) identifying the relevant studies; (3) study selection; (4) charting the data; (5) and reporting the results. Reporting of the review adheres to the Preferred Reporting Items for Systematic Reviews and Meta-Analyses (PRISMA) – 2018 Extension for Scoping Review. (Additional file 1) [23] A protocol was developed a priori and registered in Open Science Framework on August 16, 2022, and any protocol deviations have been reported there.

### Search strategy

In collaboration with a research librarian and content experts, we developed and refined a comprehensive search strategy. The strategy combined subject headings and keywords for terms related to KMb, knowledge exchange, knowledge mobilization, cross-culture, culturally appropriate, CALD communities, and adaptation of health information and implementation science. On August 12, 2021 we searched Ovid Medline (1946-), CINAHL via EBSCOhost (1937-), PsycINFO (2002-), as well as ProQuest Dissertations & Theses Global to identify grey literature. Search results were exported to EndNote V.X7 (Clarivate Analytics) and duplicates removed before the file was provided to reviewers for screening in Microsoft Excel. The search was limited to English language, peer-reviewed studies published in academic journals from 2011 to August 2021 (given KMb was introduced and reported within health research literature around 2011). A search update was run in July 2023.

### Study selection

All published study design types and secondary evidence syntheses were included if they contained a patient, public or consumer population and created or adapted a KMb product for CALD end-users. We defined a KMb product as any health research-based product that supported decision-making to provide explicit recommendations, and/or meet knowledge needs. We excluded any studies that only performed purely linguistic translations of a KMb product or the validation of translated measurement tools/questionnaires. Health interventions without standalone KMb products for end-user decision-making were also excluded. One reviewer screened titles and abstracts of each study as “include/unsure” or “exclude” based on a priori inclusion criteria (Additional file 2). A second independent reviewer verified all studies excluded by the first reviewer. Both reviewers performed a pilot screen where they independently screened 10% of the studies to assess consistency. Two independent reviewers reviewed the full-text of each included study from the primary screening. When agreement on a citation or full-text could not be reached between two reviewers, a third senior reviewer was consulted for resolution.

### Data extraction

The following details were extracted from each study: publication characteristics, study design, population, KMb product description, methods of creation or adaptation, stakeholder (defined within as “individuals, organizations or communities that have a direct interest in the process and outcomes of a project, research or policy endeavor”) [24] engagement processes, KMb product evaluation processes, and reflections from researchers. Data were collected using a standardized form by one reviewer and verified by a second reviewer. Any discrepancies were resolved through a third-party decision.

### Data analysis

We performed a narrative synthesis, guided by a qualitative content analysis approach [25] to summarize the quantity, content, and coverage of the evidence including summary statistics on studies examining the different ways of creating or adapting culturally relevant KMb products. Processes for creation or adaptation were mapped into five categories (Product creation, Literature search, Stakeholder engagement, Resources utilized, Evaluation) representing different methods reported throughout the literature.

Cultural adaptations were categorized into two broad groups, surface and deep structure [12, 26]. Surface structure involved coordinating materials and messages to observable characteristics of the target end-user (i.e., imagery, sounds, backgrounds, clothing, etc.). Deep structure involved contextualizing the social, historical, environmental, and psychological features of the proposed end-user group.

Bammer’s iS2 version [16] of the IAP2 Spectrum of Public Participation was used to classify levels of stakeholder (including end-user) engagement in the research approach/design across each study [27, 28]. The i2S is a spectrum of engagement across five stages: 1) Inform (e.g. informing stakeholders of health information and research processes), 2) Consult (e.g. researchers obtain feedback on research [i.e. recruitment processes, community engagement, tool topic], 3) Involve (e.g. researchers work directly with stakeholders to ensure their concerns or needs are considered in the research), 4) Collaborate (e.g. researchers develop equal partnerships for undertaking the research [i.e. co-designing a research study]), 5) Support (e.g. researchers support stakeholder in designing and implementing desirable research and dissemination methods) [16]. In moving from ‘inform’ to ‘support’ stakeholders have increasing influence on the research. We acknowledge that the term stakeholder may inadvertently feed into a colonialist mentality by perpetuating colonization and re-traumatization. A published model was used to define stakeholder engagement at various levels for this project and thus this term is used throughout the manuscript.

Additionally, end-user engagement in the creation or adaptation of the KMb product was mapped, based on their input into processes related to content, design, evaluation and dissemination.

## Results

### Search results

**Fig. 1:**
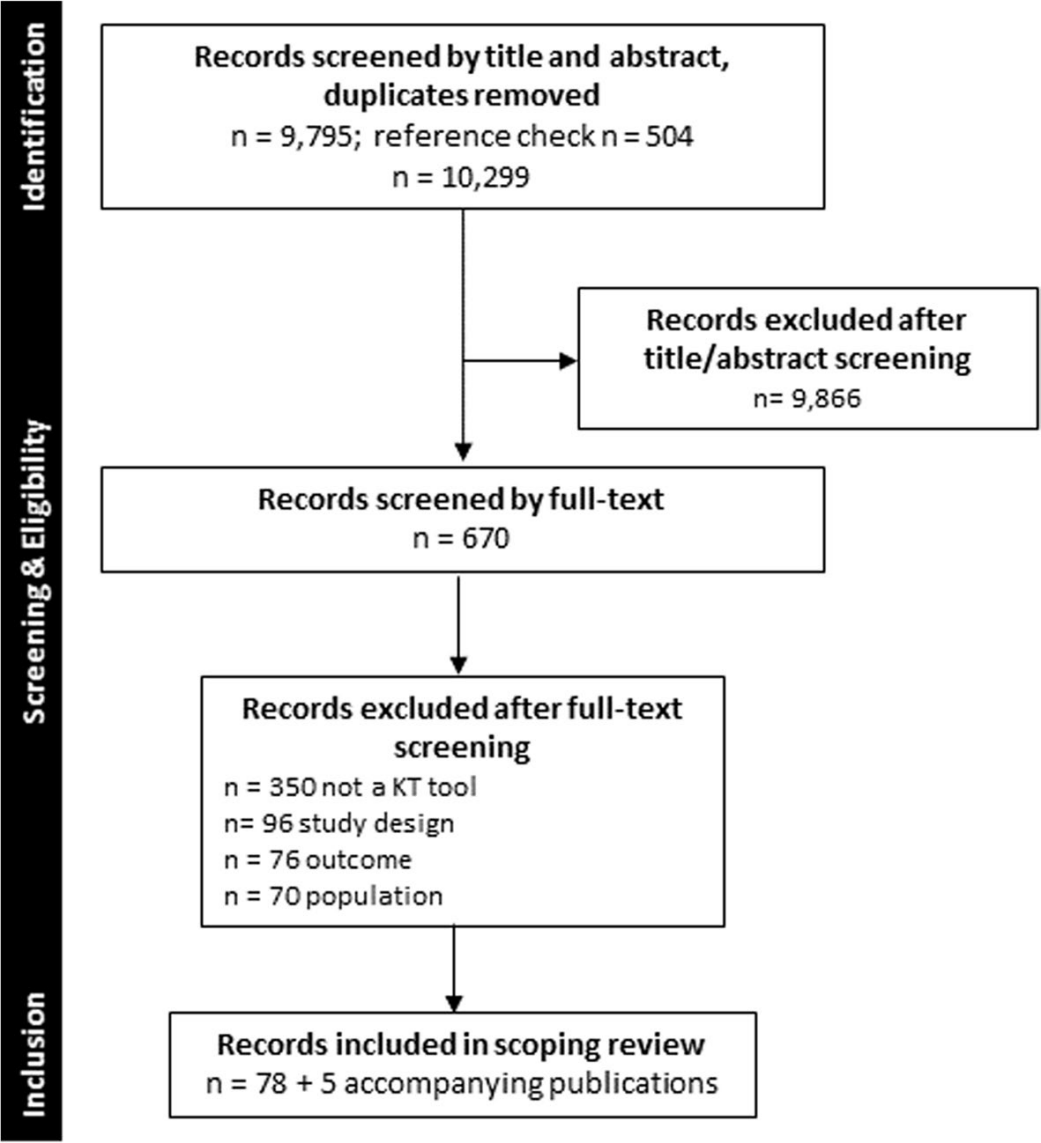
PRISMA Flow Diagram

The search strategy (Additional file 3) captured 10, 299 studies after removing duplicates. Of these, the full-text of 670 were reviewed and 78 met eligibility criteria and were included in the review. The PRISMA flow diagram (Fig. 1) provides a detailed outline of the screening and selection process.

### Study characteristics

Table 1 provides a summary of the included studies’ characteristics with detailed characteristics in Additional file 4. The majority of studies were from USA (50%, *n* = 39) and Canada (10%, *n* = 7), with three (4%) studies each from Australia and United Kingdom and two (3%) studies each from Netherlands, Portugal and Tanzania. Most of the studies used qualitative methods (78%, *n* = 61) followed by mixed methods (14%, *n* = 11) and quantitative methods (8%, *n* = 6). Forty-nine (63%) reported the age of participants, most (*n* = 45, 92%) focused on adult populations and four (8%) on adolescents.

**Table 1:** Summary of article characteristics

Study Characteristic	Number of Studies (*N* = 78)
Country	USA = 39
	Australia = 10
	Canada = 7
	United Kingdom = 3
	Netherlands = 2
	Portugal = 2
	Tanzania = 2
	Single article from each of the following: Argentina, Brazil, China, Denmark, Egypt, Guatemala, Israel, New Zealand, Norway, South Africa, Sweden, Thailand, United Arab Emirates
Study Design	Qualitative = 60
	Mixed methods = 12
	Quantitative = 6
Study Methods	Interviews = 35
	Focus group / group discussion / workshops = 33
	Surveys = 19
	Trials = 7
	Participatory / co-design research = 4
	Online correspondence = 3
	Social media / crowdsourcing = 2
	Yarning / storytelling = 2
	Situational diagnosis = 1
Language	English = 28
	Spanish = 18
	Mandarin/Cantonese = 7
	Arabic = 4
	Portuguese = 3
	Swahili = 2
	Urdu = 2
	Tiwi = 2
	Somali = 2
	Tagalong = 2
	Korean = 2
	Kiswahili = 2
	• Single article for each of the following: Dutch, Hawaiian, Italian, Japanese, Norwegian, Punjabi, Russian, Samoan, Swedish, Thai, Turkish, Vietnamese • Canadian Indigenous: Murrinh-Patha, Yolngu Matha, Kriol, Warlpiri, Central Arrernte, Pitjantjatjara • Aboriginal and Torres Strait Islander: Chuukese, Chamorro, Ndjébbana, Djambarrpuyngu, Kriol, Western Arrente, Anindilayka, Kunwinku, Marshallese, Ilocano, te reo (Maori language) • East / South African: Tigrinya, Amharic, Xhosa • South American: K’iche, Kaqchikel
Race	Hispanic / Latino = 18
	Indigenous = 12
	Black = 9
	East Asian = 7
	White = 5
	Middle Eastern = 4
	South Asian = 4
	Tanzanian = 3
	Filipino = 2
	Taiwanese = 2
	Single article for each of the following: African, Asian Australian, Brazilian, Guatemalan, Hawaiian, Moracin, Pacific Islander, Portuguese, South African, Turkish, Vietnamese
Conditions	Cancer related = 14 Childhood related = 12 Mental health related = 7 COVID related = 5
	Diabetes related = 5
	Organ donation = 4
	Health education / information = 4
	Diet / physical health = 4
	Dementia = 4
	Cardiovascular disease = 3
	Human papilloma virus = 3
	Prophylaxis = 2
	Smoking = 2
	Arthritis = 2
	Single article for each of the following: Autism spectrum disorder, Consanguineous marriage, Emergency care, HIV prevention, Incontinence, Lupus, Peripheral intravenous catheterization, Sexual health, Substance use
Target End Users	Patients = 39
	Public = 25
	Parents = 11
	Single article for each of the following: Caregivers, Children, Families

Abbreviations: *COVID* Coronavirus, *HIV* Human Immunodeficiency Virus, *USA* United States of America

End-user groups varied across the included studies, with Latinx Spanish speakers (21%, *n* = 16) as the most frequently reported cultural communities served. Seventeen (22%) studies undertook cultural adaptations into more than one language. Health topics of the KMb products included: cancer (15%, *n* = 12), mental health conditions (6%, *n* = 5), COVID topics (6%, *n* = 5), diabetes (5%, *n* = 4), organ donation (5%, *n* = 4), and dementia (5%, *n* = 4).

Forty-nine (63%) created digital KMb products, twenty-four (31%) created non-digital products and five (6%) created both a digital and non-digital version of the same KMb product. Digital KMb products (63%, *n* = 49) included Internet-based tools such as videos/animations/infographics (45%, *n* = 22), eHealth websites (14%, *n* = 7), mobile-based tools such as health applications (29%, *n* = 14), tailored text messaging campaigns (14%, *n* = 7), and patient decision aids (6%, *n* = 3). Non-digital formats included booklets/leaflets/pamphlets (79%, *n* = 19) and patient decision aids (21%, *n* = 5).

### Creation or adaptation processes

Creation or adaptation processes are mapped out in Table 2, and further details for each of the processes are outlined in Additional file 5. Each included study followed a unique process for creating or adapting a KMb product for CALD communities. Fifty-four (69%) reported the processes of creating a new KMb product, while twenty-four studies (31%) outlined their process for adapting a pre-existing KMb product for a specific end-user group. Thirty-eight studies (49%) reported a preparatory information gathering process, where literature reviews (68%, *n* = 26), systematic/scoping reviews (16%, *n* = 6), and conversations with community agencies or experts (8%, *n* = 3), provided background to direct creation of the KMb product.

**Table 2:** Processes used for creating or adapting of KMb products

Author Year Country	KMb Product Creation/Adaptation Process							
	**Product Creation**				**Literature/product search**	**Stakeholder engagement/end-user involvement**^**a**^	**Resources utilized**	**Evaluation**
	Type		Format					
	*Created*	*Adapted*	*Digital*	*Non-digital*				
Abascal-Miguel 2022 [29] Guatemala	✓		✓		NR	Community members; Key informants Interviews	Production talent; Translation support	Survey
Abbas-Dick 2018 [30] Canada	✓		✓		Information gathering	Parents / Guardians; Steering committee Interviews	Education professionals; Creative or visual designers; Healthcare providers; IT support	Survey
Alexandrou 2021 [31] Sweden		✓	✓		NR	Government/health organizations; Healthcare providers Group sessions; Collaborations	Healthcare providers; Translation support	New study conducted or underway
Ali 2019 [32] UK	✓			✓	Community networking	Community members Group sessions	Community members or organizations; Creative or visual designers; Healthcare provider; Research or content experts; Translation support	NR
Arnold 2011 [33] USA	✓			✓	NR	Community members; Healthcare providers Group sessions	Creative or visual designers	Survey
Avila 2023 [34] USA		✓	✓	✓	NR	Educators; Healthcare providers Review and revision of materials	Education professional; Healthcare providers; IT support; Translation support	Assessment tool
Baptista 2020 [35] Portugal		✓		✓	Information gathering; Systematic review of literature/guidelines	Members of target audience Interviews	Translation support	NR
Best 2012 [36] USA	✓		✓	✓	NR	Members of target audience Group sessions	NR	New study conducted or underway
Bilbrey 2018 [37] USA	✓			✓	Information gathering; Review of the literature/guidelines	Healthcare providers; Members of target audience Group sessions	NR	NR
Blazey 2023 [38] USA	✓		✓		Review of the literature/guidelines	Community members; Key informants Group sessions	Community members or organizations	Product testing
Cabassa 2012 [39] USA	✓		✓		Review of the literature/guidelines	Community members; Healthcare providers Group sessions	Community members or organizations; Creative or visual designers; Education professionals; Healthcare provider; Production talent	New study conducted or underway
Caplan 2020 [40] USA		✓	✓		Review of the literature/guidelines	Healthcare providers; Members of target audience NR	Creative and visual designer; Education professionals; Healthcare providers	Stakeholder feedback
Celentano 2021 [41] USA	✓			✓	Review of the literature/guidelines	Members of target audience Group sessions	Community members or organizations	Stakeholder feedback
Chang 2021 [42] Australia		✓		✓	NR	Healthcare providers; Members of target audience Group sessions	Translation support	Stakeholder feedback
Crouse 2023 [43] Australia	✓		✓		Review of the literature/guidelines	NR	Research or content experts	Group sessions
Cunningham-Erves 2022 [44] USA	✓		✓		Review of the literature/guidelines	Community members; Key informants Interviews	Healthcare providers; Research or content experts	Group sessions
Drago 2018 [45] USA	✓			✓	Review of the literature/guidelines	Parents/guardians Interviews	Translation support	Group sessions
Drenkard 2022 [46] USA	✓		✓		NR	Healthcare providers; Key informants; Members of target audience Review and revision of materials	Creative or visual designers; Healthcare providers	Online analytics; Surveys
DuPlessis 2022 [47] South Africa	✓			✓	Review of the literature/guidelines	Steering committee Collaborations	Creative or visual designers; Research or content experts; Translation support	NR
Elliott 2022 [11] Canada		✓	✓		NR	Members of target audience Collaborations	Creative or visual designers; Translation support	Surveys Interviews
Glennie 2022 [48] Australia	✓		✓		NR	Community members NR	Creative or visual designers; Production talent; Promotional support	Online analytics
Gordon 2015 [49] USA	✓		✓		Observation of tasks/sessions	Community members; Members of target audience Group sessions	Creative or visual designers; Education professionals; Healthcare providers; Research or content experts; Translation support	Group sessions; Product testing
Grasaas 2019 [50] Norway		✓	✓		NR	Key informants; Members of target audience Review and revision of materials	Research or content experts; Translation support	Product testing; Stakeholder feedback; Surveys
Grinker 2015 [51] USA		✓		✓	NR	Educators; Healthcare providers Interviews; Group sessions	Education professionals; Healthcare providers	NR
Guttman 2013 [52] Israel	✓		✓	✓	NR	Community members; Steering committee Interviews; Group sessions	Healthcare providers	NR
Hainsworth 2022 [53] UK	✓		✓		NR	Members of target audience Group sessions	NR	Program usage or knowledge uptake
Hall 2022 [54] Netherlands	✓			✓	Expert knowledge/experience	Community members; Members of target audience; Steering committee Group sessions	Healthcare providers	Product testing
Hamdiui 2021 [55] Netherlands	✓		✓		NR	Members of target audience Group sessions	Healthcare providers; Production talent; Research or content experts	New study conducted or underway
Harvey 2011 [56] USA	✓			✓	Information gathering; Observation of tasks/sessions	Healthcare providers; Members of target audience Interviews	Creative or visual designers; Healthcare providers; Translation support	Assessment tools
Hashim 2013 [57] UAE	✓			✓	NR	Community members; Key informants; Members of target audience Group sessions	Research or content experts	NR
Hempler 2015 [58] Denmark	✓		✓		Observation of tasks/sessions	Healthcare providers; Key informants; Members of target audience Group sessions; Interviews	Education professionals; Healthcare providers; Research or content experts	NR
Hodge 2012 [59] USA	✓		✓	✓	NR	Key informants; Members of target audience Group sessions	Translation support	Product testing; Surveys
Hong 2022 [60] USA	✓		✓		NR	Community members; Healthcare providers; Key informants; Members of target audience Group sessions	Community members or organizations; Healthcare providers; IT support	NR
Jameel 2023 [61] Australia	✓			✓	Review of the literature/guidelines	Steering committee Group sessions	Creative or visual designers; Healthcare providers	NR
Jiang 2021 [62] USA		✓	✓		NR	Members of target audience Group sessions; Interviews	Community members or organizations; Healthcare providers	Stakeholder feedback; Surveys
Jiang 2022 [63] USA	✓		✓		NR	Members of target audience Review and revision of materials	Research or content experts	NR
Kandasamy 2022 [64] Canada	✓		✓		NR	Community members; Members of target audience Collaborations	Healthcare providers; Production talent; Promotional support; Research or content experts	Program usage or knowledge uptake; Surveys
Kayler 2023 [65] USA	✓		✓		Review of the literature/guidelines	Community members; Healthcare providers; Members of target audience; Steering committee Interviews	Community members or organizations; Creative or visual designers; Production talent; Education professionals; Healthcare providers; Translation support	Surveys; Product testing
Kerr 2021 [66] USA	✓		✓		NR	Community members; Members of target audience Group sessions	Translation support; Promotional support	NR
Ko 2014 [67] USA		✓	✓		Review of the literature/guidelines	Key informants; Members of target audience Group sessions	Production talent	NR
LaMonica 2022 [43] Australia	✓		✓		Review of the literature/guidelines	Educators; Government/health organizations; Key informants NR	Education professionals; Healthcare providers; IT support; Research or content experts; Translation support	Product testing; Stakeholder feedback
Lee 2019 [68] USA	✓		✓		NR	Community members; Members of target audience Group sessions	Community members or organizations; Healthcare providers; IT support	NR
Leiter 2023 [69] USA		✓	✓		Systematic review of literature/guidelines	Key informants; Members of target audience Interviews	Healthcare providers; IT support; Production talent; Research or content experts	Group sessions
LeLaurin 2022 [70] USA	✓		✓		Review of the literature/guidelines	Healthcare providers; Key informants Review and revision of materials	Education professionals; Healthcare providers; IT support; Research or content experts; Translation support	Product testing
Lemon 2022 [71] Australia	✓		✓		NR	Community members; Government/health organizations Review and revision of materials	Community members or organizations; Production talent	NR
Li 2012 [72] USA	✓		✓		Expert knowledge/experience; Review of the literature/guidelines	Healthcare providers Review and revision of material	Healthcare providers	Stakeholder feedback
Liu 2021 [73] China/Canada	✓		✓	✓	Systematic review of literature/guidelines	Healthcare providers Review and revision of materials	Education professionals; Healthcare providers; Research or content experts; Translation support	Assessment tool
Maertens 2017 [74] USA		✓	✓		NR	Steering committee Group sessions	Community members or organizations	New study conducted or underway
Malamsha 2021 [75] United Republic of Tanzania	✓		✓		NR	Parents/guardians Group sessions	IT support; Research or content experts	Group sessions; Product testing; Surveys
Martinez 2023 [76] USA	✓			✓	Review of the literature/guidelines	Parents/guardians Review and revision of materials	Research or content experts	Stakeholder feedback
Materia 2020 [77] USA	✓		✓		Review of the literature/guidelines	Steering committee NR	IT support; Translation support	NR
Mathieson 2012 [78] New Zealand		✓		✓	Review of the literature/guidelines	Healthcare providers; Members of target audience Interviews	Creative or visual designers; Research or content experts	Surveys
Mauka 2021 [79] Tanzania	✓		✓		Review of the literature/guidelines	Members of target population Group sessions	Healthcare providers; Research or content experts	Online analytics
McFarlane 2021 [80] USA	✓		✓		NR	Members of target audience Review and revision of materials	Creative or visual designers; IT support; Research or content experts	Group discussions; Stakeholder feedback
Meherali 2021 [81] Canada	✓		✓		Systematic review of literature/guidelines	Parents/guardians Interviews	Creative or visual designers; Translation support	Stakeholder feedback; Surveys
Montague Lecturer 2022 [82] UK	✓		✓		NR	Members of target audience Interviews	Community members or organizations; Translation support	Group sessions; Stakeholder feedback
Norris 2021 [83] USA		✓	✓		NR	Educators Group sessions	Research or content experts	Group sessions; Stakeholder feedback
Pathak 2021 [84] USA	✓		✓		Review of the literature/guidelines	Members of target audience NR	NR	Product testing
Payan 2020 [85] USA	✓			✓	Review of the literature/guidelines	Community members; Key informants Review and revision of materials	Healthcare providers; Research or content experts	Program usage or knowledge uptake
Povey 2022 [86] Australia		✓	✓		Review of the literature/guidelines	Educators; Healthcare providers; Members of target population Group sessions	IT support; Translation support	New study conducted or underway
Quintana 2022 [87] Argentina	✓		✓		NR	Community members Group sessions	Community members or organizations; Creative or visual designers	Online analytics; Surveys
Rami 2018 [88] Egypt		✓		✓	Review of the literature/guidelines	Members of target audience NR	Community members or organizations	Product testing
Santos 2021 [89] Brazil		✓		✓	Systematic review of literature/guidelines	Healthcare providers NR	NR	Stakeholder feedback
Sharpe 2013 [90] USA	✓			✓	Review of the literature/guidelines	Steering committee Interviews	Creative or visual designers; Healthcare providers	Group sessions
Songtaweesin 2021 [91] Thailand		✓	✓		NR	Healthcare providers; Members of target audience Review and revision of materials	Creative or visual designers; Education professionals; Healthcare providers; Production talent	Group sessions; Stakeholder feedback
Stanley 2018 [92] USA		✓		✓	NR	Community members; Members of target audience Group sessions	Community members or organizations; Education professionals	NR
Teles 2021 [93] Portugal	✓		✓		Review of the literature/guidelines	Government/health organizations NR	Translation support	Stakeholder feedback
Tolentino 2022 [94] USA	✓		✓		NR	Community members; Members of target audience NR	Community members or organizations	NR
Umaefulam 2022 [95] Canada		✓		✓	NR	Members of target audience NR	Research or content experts	Group sessions; Stakeholder feedback
Valenzuela-Araujo 2021 [96] USA		✓	✓		NR	Member of target audience Group sessions	Creative or visual designers	Stakeholder feedback
van der Steen 2013 [97] Canada		✓		✓	NR	Member of target audience Group sessions	Healthcare providers; Research or content experts; Translation support	Conducted a new or existing study
Van Son 2014 [98] USA		✓		✓	NR	Members of target audience; Steering committee Group sessions	Education professionals; Healthcare providers; Translation support	Stakeholder feedback; Surveys
Versteegh 2022 [99] Australia		✓	✓		NR	Healthcare providers; Members of target audience Review and revision of materials	Healthcare providers; IT support; Research or content experts; Translation support	Program usage or knowledge uptake
Wall 2022 [100] USA	✓		✓		NR	Community members; Steering committee Interviews	Community members or organizations; Creative or visual designers; Healthcare providers; Promotional support	Conducted a new or existing study; Stakeholder feedback
Wright 2023 [101] Canada	✓		✓		NR	Steering committee Group sessions	Creative or visual designers; Production support	NR
Wu 2021 [102] Australia	✓			✓	Systematic review of literature/guidelines	Community members Review and revision of materials	Translation support	Stakeholder feedback
Yeager 2022 [103] USA	✓		✓		NR	Steering committee Group sessions	Community members or organizations; Healthcare providers; Production talent	Program usage or knowledge uptake; Surveys
Zerafa 2022 [104] Australia	✓		✓		NR	Members of target audience NR	Community members and organizations	NR
**Legend**								
**Product Creation**		**Created**				• Creation of a new KT product		
		**Adapted**				• Adaptation of existing KT product		
		**Digital**				• Social media (campaign, FaceBook, Short Messaging Service, ect.) • eHealth or mHealth resource (any non-mental health electronic health resource) • Radio • Fotonovela (electronic Latinx soap opera) • Animation (whiteboard, comic) • Video (CD ROM, Internet-based)		
		**Non-digital**				• Leaflet / Booklet / Pamphlet • Printed decision aid • Printed health messages, information, promotional materials		
**Literature/Product Search**		**Community networking**				• Consultation with community organizations / networks		
		**Expert knowledge/experience**				• Contact with researchers / professionals with topic experience		
		**Information gathering**				• Compiling resources on general / health specific topics • Review of similar products / decision aids / statistics		
		**Observation of tasks / sessions**				• Inspired by educational sessions • Systematic observation of patients at clinic • Observation of individual education session		
		**Review of the literature/guidelines**				• Internet based search • Literature review • Narrative or descriptive review		
		**Systematic review of literature/guidelines**				• Systematic, scoping or rapid review		
**Stakeholder engagement/end-user involvement**		**Community members**				• Business owners, pastors		
		**Educators**				• All levels of grade teachers, special education		
		**Government and health organizations**				• Swedish National Dental Health Agency		
		**Healthcare providers**				• Nurses, clinicians, family physicians, and specialists		
		**Key informants**				• Leaders, politicians, researchers, academics, experts in content area		
		**Members of target audience**				• E.g., Men with prostate cancer		
		**Parents and guardians**				• Mothers, fathers		
		**Steering Committee**				• Working groups and advisory boards • Consisting of a diverse group of individuals		
		**Types of engagement**				• Group sessions: focus groups, workshops, discussion groups/panels • Interviews: structured or semi-structured • Collaborations: with government/industry; consultations, end-users • Review and revision of materials: independent feedback given on KMb tool		
**Resources utilized**		**Community members and organizations**				• Businesses, religious groups		
		**Creative and visual designers**				• Graphic designer; animator		
		**Education professionals**				• Teachers, Lactation specialists		
		**Healthcare providers**				• Doctors, nurses, specialists		
		**IT support**				• Web developers, mobile application developers		
		**Production talent**				• Actors, producers, makeup artists, editors		
		**Promotional support**				• Advertising firms		
		**Research or content experts**				• Government officials; academics; research coordinators; public health support		
		**Translation support**				• Language translation and interpretation		
**Evaluation**		**Assessment tools**				• Standardized or validated tool		
		**Group sessions**				• Workshop based discussions and feedback		
		**New study conducted or underway**				• RCT; qualitative or quantitative methods		
		**Online analytics**				• Website visits or metrics; social media views/uses		
		**Product testing**				• Working prototype usability testing; usability of mobile App; Pilot testing		
		**Program usage or knowledge uptake**				• Increase in program attendance based on some type of decision aid		
		**Stakeholder feedback**				• Participants from creation phase or from a target audience, advisory members		
		**Surveys**				• Online or paper-based feedback through structured or open-ended questions; satisfaction questionnaires		

*IT* Information technology, *KMb* Knowledge mobilization, *KT* Knowledge translation, *NR* Not reported

^a^Stakeholders include end-users, and engagement may have occurred at different levels across different stakeholder groups (e.g. clinicians, end-users) and/or across different aspects of a project

### Stakeholder engagement in research

Twenty eight studies (36%) engaged stakeholders at varying levels in the approach to the research project, or stakeholders gave input on the research methods and study design. Most commonly, stakeholders were engaged at the level of Involve (*n* = 17, 61%), followed by Consult (*n* = 14, 50%), Collaborate (*n* = 10, 36%), and Inform (*n* = 2, 7%). Three studies (11%) engaged stakeholders at the highest level of engagement, Support. All three studies worked in partnership with American Indian or Indigenous communities, in which community-based research methods were used.

### End-user engagement in KMb product creation or adaptation

Seventy-five studies (96%) involved end-users specifically in the creation or adaptation of the KMb product. End-users were involved in providing input on content (*n* = 64, 85%) and design (*n* = 52, 69%) features. Input was gathered through a mix of focus group sessions (*n* = 24), one-on-one interviews (*n* = 31), surveys (*n* = 7), workshops (*n* = 7) and via community advisory groups (*n* = 36). Over half the studies (*n* = 44, 59%) reported involving end-users in the evaluation of the KMb product. This included seeking feedback from end-users via surveys (*n* = 14, 32%), focus groups (*n* = 5, 11%) as well as formal evaluation studies (*n* = 9, 20%). Of these, 40 (91%) studies reported the results of their evaluation. Assessments included usability testing, acceptability, cultural appropriateness, esthetics, and knowledge gained through use of the KMb product. However, none of the studies included a structured assessment of end-user engagement and involvement when adapting or creating the KMb product. Twenty studies (27%) reported that end-users were involved in the dissemination of the created or adapted KMb resource. Thirteen studies (65%) reported end-users provided suggestions on where and how to disseminate, and in 11 studies (55%) end-users helped with dissemination of the KMb resource.

### Resources utilized

Most studies (*n* = 74, 95%) reported the various human resources they utilized during their creation or adaptation process. The involvement of healthcare providers was the most prevalent (*n* = 35, 47%), followed by translation support (*n* = 27, 36%), research or content experts (*n* = 25, 34%) and creative or visual designers (*n* = 22, 30%). Two studies used specialists to moderate their process, one study [32] had a participatory design expert to assist with guiding exercises and another study [57] used an experienced moderator to lead their discussion sessions.

### Approach and type of cultural adaptation

Details regarding the type of cultural adaptations used and involvement of end-users is presented in Table 3. Six studies (8%) cited using a CBPR [18] approach, and twenty-five studies (32%) reported using a framework, theory or model to guide their creation or adaptation efforts. All studies utilized surface structure cultural adaptations and most (85%, *n* = 66) included deep structure adaptations in their creation or adaptation processes. To achieve deep structure contextualization authors most commonly consulted specific end-user populations (e.g. clinical populations [14%, *n* = 9], community members [30%, *n* = 20]) through focus groups (11%, *n* = 7), and assessed various cultural contexts (18%, *n* = 12).

**Table 3:** Type of cultural adaptation and end-user involvement in KMb creation or adaptation

Author Year Country	Type of Theoretical Framework/ Theory/ Model	Adaptation		End-user Involvement				Researcher Experiences
		**Surface**	**Deep**	**Content**	**Design**	**Evaluation**	**Dissemination**	
Abascal-Miguel 2022 [29] Guatemala	NR	- Linguistic translation and local voice narration	- Key informant interviews and community focus groups used understand vaccination barriers, including access, supply, trust and fear	✓	✓	✓	✓	NR
Abbas-Dick 2018 [30] Canada	NR	- Changed layout of information - Changed mode of delivery	- Improved cultural relevance of the content and communication based on specific cultural barriers identified	✓	✓	✓	NR	NR
Alexandrou 2021 [31] Sweden	NR	- Linguistic translation of app to Somali, Arabic and English - Addition of audio–video files of content in Somali and Arabic	NR	✓	✓	NR	NR	NR
Ali 2019 [32] UK	Social Marketing [105] Representational Artefacts [106]	- Changed the layout of information	- Assessed cultural relevance and application with community members and adapted visuals and language accordingly	✓	✓	NR	NR	
Arnold 2011 [33] USA	NR	- Changed visuals to appear more consistent with cultural images	- Participants modified the voices and language to express their cultural values and traditions	✓	✓	✓	✓	NR
Avila 2023 [34] USA	NR	- 2 step linguistic translation (USA Spanish) - changes to graphics and layout	- Cultural review done to ensure sociocultural aspects of patient’s lived experiences were reflected in materials	NR	NR	NR	NR	
Baptista 2020 [35] Portugal	Translation is not enough – European Centre for Disease Prevention and Control [107]	- Forward and back linguistic translation - Changed visuals to appear more consistent with cultural images	-Changed the context to match the healthcare provisions in Portugal - Assessment of barriers to healthcare access for Portuguese men	✓	✓	NR	NR	NR
Best 2012 [36] USA	Fractured Paradigm of Media Framing [108] Religiosity, spirituality, and the design of health communication message and interventions [109]	- Changed visuals to appear more consistent with cultural images	- Identified and prioritized the most important spiritual elements to be included in messages targeted to African American women	✓	NR	✓	NR	NR
Bilbrey 2018 [37] USA	NR	- Changed visuals to appear more consistent with cultural images	- Community conversations guided content creation of brochures on brain donation	✓	✓	NR	NR	NR
Blazey 2023 [38] USA	Social cognitive theory [110] Culturally informed conceptual framework [111]	- Visual appearance and inclusivity of images and infographics changed	- Target population’s collective wisdom, self-sacrifice, and experiential knowledge were used to inform creation of resources	✓	✓	✓	NR	NR
Cabassa 2012 [39] USA (Sanchez 2021)	Illness perception theory [112]; reasoned action theory [113]; Entertainment-Education communication strategy [114]	- Linguistic translation of *fotonovela* content into Spanish - F*otonovela* independently reviewed by community members (feedback on style, content length and design)	- Based on multi-stakeholder input, characters were created that resembled the target population ad could serve as positive role models	✓	✓	NR	NR	
Caplan 2020 [40] USA	Information system research framework [115]	- Linguistic translation	- Culture, and social characteristics of the Dominican population were embedded—Elements of religious gratitude were added to the program	✓	✓	NR	NR	NR
Celentano 2021 [41] USA	Health Belief Model [116] and Theory of Reasoned Action [117]	- Content was translated from English into Somali, Amharic, and Tigrinya -Photos of adolescents that resembled members of East African communities were used	- Comic book mock-up was reviewed for cultural relevance and socio-cultural beliefs by East African community members	✓	✓	✓	NR	NR
Chang 2021 [42] Australia	International Patient Decision Aids Standards [118]	- Forward and back linguistic translation	- Conceptual equivalence and cultural adaption undertaken by consulting experts to assess content of translation, cultural appropriate modification and the booklet presentation	NR	NR	✓	NR	NR
Crouse 2023 [43] Australia	NR	- Linguistic translations	- A cultural framework is created collaboratively with the research team and a nominated country-specific expert	✓	✓	✓	NR	NR
Cunningham-Erves 2022 [44] USA	Theory of reasoned action [119]; Health Belief Model [120]; 5 strategies to achieve cultural appropriateness [121]	- Images and graphics iteratively revised to depict culture	- Community partners ensured messaging represented top vaccination concerns and was culturally appropriate	✓	✓	NR	NR	NR
Drago 2018 [45] USA	NR	- Linguistic translation	- Themes from birthing experiences of the target population were included	✓	NR	✓	NR	NR
Drenkard 2022 [46] USA	NR	- Linguistic translation	- Adaptations conducted to meet audience's health literacy and cultural values	✓	NR	✓	✓	
Du Plessis 2022 [47] South Africa	NR	- Translation and back translation -Images changes to reflect various cultures	- Cultural eating habits (cooking methods, food availability) were taken into consideration for messaging	✓	✓	NR	✓	
Elliott 2022 [11] Canada	Ecological Validity Framework [20]	- Forward-back linguistic translation of narration and video text - Character visuals adapted to represent Filipino community more accurately	NR	NR	✓	✓	✓	NR
Glennie 2022 [48] Australia	Local communication strategies [122, 123]	- Linguistic translation	- Resources created by members of community	✓	✓	NR	NR	NR
Gordon 2015 [49] USA	Gagne’s Conditions of Learning Theory [124] and Bandura’s Social Cognitive Theory [110]	-Linguistic translation - Used colors to coincide with Hispanic sensibilities - Pictures of traditional Hispanic food	- Focus groups were used to discuss the cultural beliefs and myths about kidney donation - Content delivery such as through immersive multimedia and telenovelas	✓	✓	✓	✓	NR
Grasaas 2019 [50] Norway	Translation and Cultural Adaptation of Patient Reported Outcomes [125]	- Forward linguistic translation - Replaced names organizations to be culturally relevant	- Involved end-users to assess the content for relevance	✓	NR	✓	NR	NR
Grinker 2015 [51] USA	Cultural Consensus Theory [126]	- Changed language to that preferred by end-users	- Mothers, teachers, and clinicians provided context for mental health conditions	✓	NR	NR	NR	NR
Guttman 2013 [52] Israel	NR	- Forward and back linguistic translation - Changed visuals to appear more consistent with cultural images	- Solutions were based on the actual stories and recommendations of members of the community	✓	NR	NR	NR	NR
Hainsworth 2022 [53] UK	NR	NR	- Resources co-created by members of community	✓	NR	NR	✓	
Hall 2022 [54] Netherlands	Social cognitive theory [127] and Intervention mapping [128]	- Linguistic translation	- Revisions of the artwork were made with respect to cultural and religion	✓	✓	✓	NR	
Hamdiui 2021 [55] Netherlands	Entertainment–Education communication strategy [114]	- Linguistic translation Turkish, Moroccan‐Arabic, and ‐Berber	- Culturally appropriate background music used - Type and content of the storyline, recruitment and type of actresses, and the setting of the video was created with input from community members	✓	✓	✓	NR	NR
Harvey 2011 [56] USA	Precede-Proceed Model [129]	- Forward linguistic translation - Changed visuals to appear more consistent with cultural images	NR	✓	✓	NR	✓	NR
Hashim 2013 [57] UAE	NR	- Forward and back linguistic translation - Changed visuals to appear more consistent with cultural images	- Focus groups were held to discuss the application of suggestions for their culture	✓	✓	NR	NR	NR
Hempler 2015 [58] Denmark	NR	- Changed visuals to appear more consistent with cultural images	- Researchers engaged with patient involvement about how dialog products work in real life	✓	✓	NR	NR	
Hodge 2012 [59] USA	NR	- American Indian cancer survivor testimonies and storytelling - Southwest American Indian Imagery incorporated throughout	- Discussion of cultural constructs in focus groups	✓	✓	✓	✓	
Hong 2022 [60] USA	NR	- Linguistic translation	- Cultural appropriateness of app was reviewed by community stakeholders - Cultural nuances taken into consideration in translation (e.g., preferred to use “the elder” rather than care partner)	✓	✓	NR	NR	NR
Jameel 2023 [61] Australia	NR	- Imagery adapted (e.g.,Wedge-tailed Eagle chosen as main character and narrator to ensure cultural relevance	NR	✓	✓	NR	NR	NR
Jiang 2021 [62] USA	Social cognitive theory [127]	- Linguistic translation	- content added to align with Vietnamese context (e.g., included practices that were relevant to Vietnamese smokers)	✓	NR	✓	NR	NR
Jiang 2022 [63] USA	Social cognitive theory [127]	- Linguistic translation	NR	✓	✓	NR	NR	NR
Kandasamy 2022 [64] Canada	NR	- Linguistic translation	- Community members involved in deciding upon visuals, colours and a culturally authentic plot for the video	✓	✓	✓	✓	
Kayler 2023 [65] USA	Bandura's self-efficacy theory [130]; Mayer’s cognitive theory [131]	- Linguistic translation - Greater variation in characters’ skin tone to broaden the multicultural aspect of the images	- To culturalize the animations to fit African American population's needs, specific design and content characteristics were included	✓	✓	✓	✓	
Kerr 2021 [66] USA	NR	- Ensured diversity of skin tone within website imagery	NR	✓	✓	NR	✓	
Ko 2014 [67] USA	Cultural and linguistic adaptation framework created for this project based on literature [132]	- translated from English to Spanish - narration by professional Mexican actor	- target population focus groups confirmed socio-cultural influences on screening	✓	✓	✓	NR	NR
LaMonica 2022 [43] Australia	Medical Research Council’s Framework for Complex Interventions [133]	- Linguistic translations	- Content adapted to the cultural context of each country valuing cultural traditions	NR	NR	✓	NR	NR
Lee 2019 [68] USA	NR	- Changed visuals to appear more consistent with cultural images	- Cultural misconceptions addressed through focus groups	✓	✓	NR	✓	NR
Leiter 2023 [69] USA	Structural Influence Model of Health Communication [134]	- Multi-step linguistic translation	- Authentic narratives and voices of Latino patients used - integrated pertinent patient quotes	✓	✓	✓	NR	
LeLaurin 2022 [70] USA	NR	- Linguistic translation - Images of Hispanic caregivers and patients	- Spanish speaking healthcare providers and caregivers reviewed materials for language and cultural appropriateness	✓	✓	✓	✓	
Lemon 2022 [71] Australia	NR	- Local community members case in videos	- Scripts used culturally appropriate language and portrayals of relationships and interactions	✓	✓	NR	NR	
Li 2012 [72] USA	NR	- Changed visuals to appear more consistent with cultural images	NR	NR	NR	✓	NR	NR
Liu 2021 [73] China/Canada	NR	- Forward and back linguistic translation	- The team carefully considered ethnocultural differences in terms of customs (including family structure), values (e.g., filial piety), and beliefs (e.g., value of traditional Chinese medicine)	NR	NR	✓	✓	
Maertens 2017 [74] USA	NR	- Linguistic translation - Iterative revisions to the logo, and color scheme	- Attitudinal, infrastructural, and cultural factors impacting decisions were incorporated	✓	✓	✓	NR	NR
Malamsha 2021 [75] United Republic of Tanzania	Game theory and creation lifecycle [135, 136]	- Story script was composed in Swahili and English languages	- Experts provided feedback on topics and sociocultural requirements for prevention education against child sexual exploitation, and the obstacles that come with it in Tanzania	✓	✓	✓	NR	NR
Martinez 2023 [76] USA	Cultural adaptations of behavioral health interventions [137]	- Pictographs included photographs reflecting matching surface structure for African American and Caucasian American families	- Adaptations based on targeted cultural domains: locus of control, acceptability, and responsibility	✓	NR	✓	NR	
Materia 2020 [77] USA	RE-AIM Framework [138], IDEAS framework for mHealth [139], Consolidated Framework for Implementation Research [140]	- Translation of SMS messages to the Xhosa language	- Community advisory board reviewed SMS library to ensure it matched cultural and social norms of participants	✓	✓	NR	NR	
Mathieson 2012 [78] New Zealand	NR	- Changed visuals to appear more consistent with cultural images	- Traditional practices were incorporated based on end-user comments	✓	✓	✓	NR	
Mauka 2021 [79] Tanzania	Information system research framework [141]	- Population-specific and sensitive language translation from English to Swahili	NR	✓	✓	NR	NR	NR
McFarlane 2021 [80] USA	NR	- Linguistic translation of resources into Spanish	- The website includes hand-drawn whiteboard animations that are embedded into various web pages and features a Hispanic female protagonist	NR	NR	✓	✓	NR
Meherali 2021 [81] Canada	NR	- Forward and back linguistic translation - Changed visuals to appear more consistent with cultural images	- Inclusion of the cultural contexts for seeking healthcare	✓	NR	✓	✓	NR
Montague Lecturer 2022 [82] UK	COM-B behaviour change model [142]	- Linguistic translations - Cultural neutrality of the graphics	- Animation was sensitive to social and cultural background as avatars were both non-gender specific and not reflective of any particular ethnic background	NR	NR	✓	NR	
Norris 2021 [83] USA	NR	- Changed language to that preferred by end-users	NR	✓	NR	✓	NR	NR
Pathak 2021 [84] USA	Social cognitive theory [143], behavior change models [142, 144]	- Linguistic translations performed following a culturally sensitive communication standard	- Messages not translated literally; rather, adapted and reframed to consider ethnic diversity and other important cultural characteristics	✓	NR	✓	NR	
Payan 2020 [85] USA	Input–output framework for constructing persuasive messages [145] and the health belief model [116]	- Linguistic translation into Spanish and back-translated by a native Spanish speaker before use	- Storyboards with different graphical representations and fictional narratives of Latinas	✓	✓	✓	NR	NR
Povey 2022 [86] Australia	NR	- Integrated Indigenous languages - Feedback on design styles, storylines, and character attributes	- Exploring and including cultural identities	✓	✓	NR	NR	
Quintana 2022 [87] Argentina	Collaborative action research method [146]	- Linguistic translation, music, colors, and general esthetics created with community input	- community members involved in creation of culturally sensitive videos	✓	✓	✓	NR	
Rami 2018 [88] Egypt	NR	- Changed language to that preferred by end-users	- Cultural contexts in Egyptian society were noted by researchers	NR	NR	✓	NR	
Santos 2021 [89] Brazil	NR	- Visuals changed to ensure skin tone matched target audience	NR	NR	NR	NR	NR	NR
Sharpe 2013 [90] USA	NR	- Changed visuals to appear more consistent with cultural images	- Continued involvement from partners directed content selection for the materials	✓	✓	✓	NR	NR
Songtaweesin 2021 [91] Thailand	NR	- Linguistic translation	- Stories and images were adapted to Thai context	✓	✓	NR	NR	
Stanley 2018 [92] USA	Cultural Adaptation of Treatments [147]	- Changed language to that preferred by end-users	- Use of culturally significant leaders for messaging and used messaging consistent with the experiences of youth	✓	✓	NR	✓	NR
Teles 2021 [93] Portugal	NR	- Linguistic translation from British English into European-Portuguese	- Various elements underwent cultural adaptation: words/ expressions; personal names of characters; resources; and references to cultural habits, customs, and traditions	NR	NR	NR	NR	
Tolentino 2022 [94] USA	NR	- Infographics translated into different languages to reach communities that did not speak English	NR	✓	✓	NR	✓	
Umaefulam 2022 [95] Canada	Informed by cultural adaptation of another shared decision making tool with Indigenous population [148]	- Simplified text, included appropriate Indigenous symbols, images and colors	- Include options on Indigenous traditional healing	✓	✓	NR	NR	NR
Valenzuela-Araujo 2021 [96] USA	NR	- Linguistic translation	- Family advisory council reviewed video for cultural appropriateness	✓	NR	✓	✓	
van der Steen 2013 [97] Canada	NR	- Forward and back linguistic translation - Changed visuals to appear more consistent with cultural images	- Changed the level of involvement of the healthcare providers to match real-word scenarios	NR	NR	✓	NR	NR
Van Son 2014 [98] USA	NR	- Changed visuals to appear more consistent with cultural images	NR	NR	NR	✓	NR	NR
Versteegh 2022 [99] Australia	NR	- Forward and back linguistic translation	- Graphics and audio adapted and approved for cultural appropriateness	NR	NR	✓	NR	NR
Wall 2022 [100] USA	Educational-entertainment models [149] and social cognitive theory [127]	-Resource tailored to individuals	- Religious, culture/knowledge, altruistic, and normative content considered	✓	✓	✓	NR	NR
Wright 2023 [101] Canada	Principles of Indigenous KT [150]	- Videos created by Indigenous mothers	- videos done by Indigenous mothers	✓	✓	NR	NR	
Wu 2021 [102] Australia	Framework of cultural sensitivity [12]	- Linguistic translation of booklets	- Chinese community evaluated cultural appropriateness	✓	✓	NR	NR	
Yeager 2022 [103] USA	NR	- Community members present in videos	- Culturally relevant information was presented in an understandable and engaging format (active voice, simplified content, and authentic messaging)	✓	✓	✓	NR	NR
Zerafa 2022 [104] Australia	NR	- Created independent of written or spoken language	- Inclusion, storytelling, transparency, empowerment and dignity were important cultural considerations	✓	✓	NR	NR	

*Abbreviations*: *AA* African American, *APP* Application, *BCS* Breast Cancer Screening, *COVID* Coronavirus, *ED* Emergency Department, *HIV* Human Immunodeficiency Virus, *HPV* Human Papilloma Virus, *KMb* Knowledge Mobilization, *KT* Knowledge Translation, *MH* Mental Health, *NR* Not Reported, *PrEP* Pre-Exposure Prophylaxis, *RCT* Randomized Controlled Trial, *RA* Rheumatoid Arthritis, *SMS* Short Message Service, *UK* United Kingdom, *USA* United States of America

Definitions: 1. *Content*: informal or formal methods (e.g. focus groups, interviews); barriers; facilitators; advice; tips; facts; storytelling; content addressing specific topics that are important to end-users; tailored content based on cultural aspects (e.g. seasonal foods, recipes); gain insight in attitudes; knowledge and information needs; readability and understanding of content;.2. *Design:* modality of tool; images; illustrations; symbols; script development; video-voice over; use of games; font; colours; links to additional content; app features (e.g., interactive functions); video length, positive voice; organization of content; ease of using the tool;3. *Evaluation:* consideration on whether end-users were involved in evaluating the final product*;*4. *Dissemination:* end-user involvement in planning or direct dissemination of the resource

✓Denotes whether end-user involvement was used and if researchers reported reflections

### Researcher reflections

Thirty-two (41%) studies included reflections from the research team on the processes for creating or adapting KMb products (See Additional File 6). Notably, researchers emphasized the importance of forming stakeholder relationships before and involving end-users throughout the research process [32], that communication between multiple stakeholder groups can be time-consuming [58], that initial positive reception to adapted products does not guarantee adherence to behavior change [88], but that the process of adapting KMb products can be rewarding for researchers (e.g. meeting end-users needs) [78].

## Discussion

This ScR provides an outline of documented processes used to create or adapt KMb products for CALD communities, highlights gaps in that literature, and provides direction for future research. As a means of addressing the needs of populations often underserved by health systems, researchers and organizations have begun specifically creating or adapting their KMb products for CALD communities. To the best of our knowledge, this is the first review to synthesize and examine literature on processes and considerations for creating or adapting KMb products. There appears to be a range of methods employed to address KMb creation for CALD groups. These methods range from original co-created KMb products with participatory frameworks (e.g. Wild et al., [10]; Telenta et al., [14]) to cultural adaptations of pre-existing KMb products [81].

Through this ScR, we identified 78 studies that reported a variety of methods for creating or adapting KMb products for CALD communities. Across the various cultural communities, modes of information delivery, and approaches/processes cited, many studies demonstrated deep structure cultural adaptation [12]. While the majority involved end-users in the creation or adaptation of the KMb resource, only 28 studies engaged stakeholders in the research approach (as per i2S model) [16].

Along with study characteristics and creation processes, we extracted information about the depth of creation or adaptation based on Resnicow’s [12] explanation of surface and deep structures of cultural sensitivity. It has been reported that gaining deep structure cultural knowledge can be a time-consuming process, largely inaccessible to outsiders to the cultural community [151]. Although it was not possible to extract information about the cultural background of included studies team members, it is likely that researchers may not identify with the end-user population of study. Researchers who are outsiders to the end-user community lack the necessary information for deep structure cultural sensitivity on their own. However, engaging with community members directly can provide insider perspectives for culturally sensitive practices.

Each phase of the i2S framework represents increasing involvement of stakeholders in research processes [16]; with the ultimate stage of *Support* representing research decisions led by end-users. The few studies that utilized this level of the i2S framework in this review potentially indicate the challenges and commitment required for this process. Studies that exemplified the *Support* phase of the i2S utilized end-user committees that were involved from early conversations about research priorities to eventual dissemination of findings. However, while it is important to note that stakeholder engagement may vary depending on project aims and resources, ongoing stakeholder engagement at the Involve, Collaborate, or Support level is essential to gain insights for deep structure cultural aspects and relevant KMb [26]. The majority of studies included in this review engaged in some form of deep structure cultural adaptation, likely due in part to some form of end-user engagement and involvement [16] reported in included studies.

This emphasis on inductive knowledge obtainment and delivery mirrors processes outlined in CBPR [152] and PAR [17] approaches. In both CBPR and PAR, the end-user from the community of study is positioned as a collaborator: someone who has autonomy in the research process as well as insider information for the community of study [17, 18, 152]. CBPR has been used as a guiding approach in health intervention literature, and may provide similar guidance for KMb product creation and adaptation [153]. Additionally, frameworks used for adapting health interventions, such as the Ecological Validity Model [20], may also offer a systematic approach to cultural adaptations of KMb products. Regardless of the framework used, researchers who choose to create or adapt KMb products for CALD communities may be well-supported by seeking deep structure cultural understandings through supportive, inductive stakeholder engagement and through involving end-users in the development of KMb resources.

A gap in the literature was around researchers’ reflections of the processes used, as well as the specific methods of KMb product evaluation. Many involved a wide range of people and skill sets, which is also potentially time consuming and costly. While many studies reported they evaluated the created or adapted KMb product for usability, few mentioned the specific tools used to assess uptake and impact. It is unknown whether some of the initial positive receptions to the adapted products reported resulted in increased knowledge or influenced behavior change or decision-making (pending the purpose of the tool). Evaluation tools should assess not only the cultural appropriateness of the developed or adapted KMb product, but also the effectiveness of the products in terms of achieving their intended purpose (e.g. increased health literacy, influenced decision making). Further, no studies reported evaluating the engagement process with their end-users. Additionally, many did not report on the practicality or feasibility of the processes used (time, resources, engagement), nor whether the product met end-users’ needs and expectations. Those that did, reflected that incorporation and balancing of opinions and feedback from different stakeholders (researchers, clinicians, end-users, community members) was difficult and time-consuming. Further, fostering collaborations between researchers and community members was resource intensive, yet many reported that establishing these partnerships was key to ensure materials were comprehensive, accessible, and appropriate for the end-users.

Future research should aim to understand the practicalities and nuances of engaging end-users and evaluating the processes to support others in this field. Furthermore, greater transparency by researchers in their adaptation processes would aid in solidifying best practice considerations for culturally adapting KMb products. Ultimately, the most successful methods used by researchers to create or adapt KMb products for CALD communities could be collated and used to form a framework for future work. Additionally, drawing on culturally targeted or tailored approaches proposed by Kreuter et al., [121] could help identify factors such as familial roles, communication patterns, belief systems, social structures and other behavioral and social characteristics within the end-user community that should be considered during tool development or adaption. A framework that integrates peripheral, evidential, linguistic, and social cultural dimensions, could then be evaluated with end-users from various cultural communities to assess its usefulness in this field [121]. However, given how nuanced and tailored KMb should be in meeting the needs of the end-user, perhaps careful planning considering meaningful engagement and being intentional about the best methods to use is key.

### Limitations

This review only included publications in English, yet other cultural creation or adaptation methods studies may be present in languages other than English. The process of defining a KMb product was iterative and largely guided by consensus discussion. The overlap between KMb products and intervention materials was difficult to navigate, particularly when studies did not thoroughly describe their intervention materials.

### Consultation

By examining the methods others have used for their creation and adaptation work, a better understanding around the key considerations when creating or adapting KMb products for culturally and linguistically diverse communities can be achieved.

A methods working group stemming from this work has been developed to drive the creation of key considerations for how to linguistically and culturally create or adapt KMb products. The methods working group is made up of researchers, cultural knowledge brokers and community members who have firsthand experience and knowledge around how to engage with diverse communities as well as co-design KMb products. By critically evaluating current adaptation practices, we intend to establish a core set of methods and considerations for creating or adapting healthcare decision-making tools for CALD communities. A driving questions behind those discussions will be: is it possible to create a KMb product that meets the needs of multiple diverse communities, or does that go against the foundational tenets of KMb (contextualization, target end-user)?

## Conclusion

This review provides information on the various processes, resources needed and levels of stakeholder engagement and end-user involvement used to create or adapt KMb products for CALD communities. While methods and processes, as well as theory or frameworks underpinning the work, varied across projects, it is clear that an important amount of time and resources is required. Significant gaps in the literature still remain surrounding how best to create or adapt culturally relevant KMb products and how to evaluate their impact, what level of engagement is needed, as well as understanding the practicalities of culturally adapting KMb products. Until an appropriate framework exists that integrates both cultural and linguistic dimensions, researchers would be well-supported by emphasizing cultural sensitivity and meaningful end-user engagement in their approaches.

The findings of this review and examples of cultural adaptation could be used to support the creation of best practice guidelines for researchers working in this field. Understanding and developing considerations for best practices will assist researchers and organizations in effectively reaching a wider population with health promotion and KMb initiatives.

### Supplementary Information

Supplementary Material 1.

Supplementary Material 2.

Supplementary Material 3.

Supplementary Material 4.

Supplementary Material 5.

Supplementary Material 6.

## Data Availability

No datasets were generated or analysed during the current study.

## Abbreviations

CALD: Culturally and linguistically diverse
CBPR: Community-based participatory research
IAP2: International Association for Public Participation
KMb: Knowledge mobilization
PAR: Participatory Action Research
ScR: Scoping review
