# Supplementary material for: Exploring methods for creating or adapting knowledge mobilization products for culturally and linguistically diverse audiences: a scoping review
Source: Arch Public Health. 2024 Jul 22;82:111. doi: 10.1186/s13690-024-01334-0 (PMC11265177; doi:10.1186/s13690-024-01334-0)
Supplement: Supplementary file 2 — Supplementary Material 2. [file 13690_2024_1334_MOESM2_ESM.docx]

**Additional file 2: Eligibility criteria**

| **Characteristic** | **Inclusion Criteria** | | **Exclusion Criteria** |
| --- | --- | --- | --- |
| Publication date | The last 10-12 years (2011 onwards, with search update) | | Prior to 2011 |
| Language | English | | Languages other than English |
| Study design | Primary studies: qualitative, quantitative, mixed methods  Secondary studies: reviews or overviews of reviews  Theses and dissertations  Opinion pieces (e.g. commentaries, editorials) published in peer-reviewed journals | | Unpublished opinions pieces (e.g. blogs, online commentaries) and manuscripts. |
| Population | Patients/public/consumers of any age (e.g. adults and children) from culturally and linguistically diverse communities.  Researchers who have worked with culturally and linguistically diverse audiences during the cultural adaptation process. | | Non-patients/public/consumers (e.g. health care provider, medical practitioner, nurse, teacher, school administrator, legislator, epidemiologist, community health worker, policy analyst).  Non-researchers who belong to majority cultural groups. |
| KMb Product | Creation or adaptation of a KMb product for culturally and linguistically diverse communities.  Defined as products presenting research-based information in user-friendly language and format(s) to potentially support decision-making (e.g. it might be information to help make the decision, or to increase knowledge [and a down-stream effect might be impact on decision-making].  E.g. patient decision aid, interactive infographic, blogshot, whiteboard animation video. | | Creation or adaptation of a *health intervention or promotion program* for culturally and linguistically diverse communities.  Intervention or promotion materials or programs that include tangible products for their target population to help implement the intervention or learn a specific skill set.  Non-health related implementation or KMb products/interventions/ strategies. |
| Outcome | Methods and processes of creating or culturally adapting KMb products related to health  Evaluations, considerations/lessons learned for creating or adapting health information for culturally and linguistically diverse communities | Processes entailing purely linguistic adaptations of health information.  Studies listing cultural adaptation, without further explanation of processes.  Studies detailing the validation of translated documents (i.e. questionnaires and assessments). | |
