# Supplementary material for: Exploring methods for creating or adapting knowledge mobilization products for culturally and linguistically diverse audiences: a scoping review
Source: Arch Public Health. 2024 Jul 22;82:111. doi: 10.1186/s13690-024-01334-0 (PMC11265177; doi:10.1186/s13690-024-01334-0)
Supplement: Supplementary file 3 — Supplementary Material 3. [file 13690_2024_1334_MOESM3_ESM.docx]

**Ovid MEDLINE(R) ALL <1946 to** July Week 2 2023**>**

**Date of search: July 16, 2023**

1 Consumer Health Information/ (4095)

2 ((consumer* or patient* or public) adj2 health information).ti,ab,kf. (2194)

3 Health Promotion/ (77181)

4 (health adj2 (promotion* or campaign*)).ti,ab,kf. (40630)

5 Health Communication/ (2815)

6 health communication.ti,ab,kf. (3921)

7 exp Patient Education as Topic/ (87448)

8 ((patient* or consumer*) adj2 education*).ti,ab,kf. (28891)

9 Translational Medical Research/ (11900)

10 ((knowledge adj3 (translat* or transfer* or mobiliz* or mobilis* or exchange or implement* or disseminat* or uptake or adopt* or application* or apply or applie*)) and (service* or tool* or campaign* or program* or resource* or product*)).ti,ab,kf. (10353)

11 ((evidence adj3 (translat* or transfer* or mobiliz* or mobilis* or exchange or implement* or disseminat* or uptake or adopt* or application* or apply or applie*)) and (service* or tool* or campaign* or program* or resource* or product*)).ti,ab,kf. (9326)

12 ((research adj3 (translat* or transfer* or mobiliz* or mobilis* or exchange or implement* or disseminat* or uptake or adopt* or application* or apply or applie*)) and (service* or tool* or campaign* or program* or resource* or product*)).ti,ab,kf. (23587)

13 ((innovat* adj3 (translat* or transfer* or mobiliz* or mobilis* or exchange or implement* or disseminat* or uptake or adopt* or application* or apply or applie*)) and (service* or tool* or campaign* or program* or resource* or product*)).ti,ab,kf. (3574)

14 ((best practice* adj3 (translat* or transfer* or mobiliz* or mobilis* or exchange or implement* or disseminat* or uptake or adopt* or application* or apply or applie*)) and (service* or tool* or campaign* or program* or resource* or product*)).ti,ab,kf. (1510)

15 ("knowledge into practice" or "knowledge to practice" or "knowledge into action" or "knowledge to action").ti,ab,kf. (5403)

16 ("evidence into practice" or "evidence to practice" or "evidence into action" or "evidence to action").ti,ab,kf. (13608)

17 ("research into practice" or "research to practice" or "research into action" or "research to action").ti,ab,kf. (12970)

18 ("innovation* into practice" or "innovation* to practice" or "innovation into action" or "innovation to action").ti,ab,kf. (251)

19 (evidence based adj2 (program* or service* or intervention* or campaign* or tool* or resource*)).ti,ab,kf. (11038)

20 (KMb adj2 (product* or tool* or intervention* or strateg* or campaign* or service*)).ti,ab,kf. (306)

21 (brochure* or booklet* or pamphlet*).ti,ab,kf,hw. (10594)

22 ((app or apps or application*) adj3 (mobile or device* or cell* or iphone* or android* or smart-phone* or smartphone*)).ti,ab,kf,hw. (46688)

23 (social media or facebook or instagram or twitter).ti,ab,kf,hw. (23959)

24 or/1-23 (367959)

25 Culture/ (33657)

26 cross-cultural comparison/ (26456)

27 Cultural Characteristics/ (16714)

28 Cultural Competency/ (5981)

29 Culturally Competent Care/ (1823)

30 cultural diversity/ (12046)

31 ((cultur* or ethnocultur*) adj5 (adapt* or translat* or modif* or tailor*)).ti,ab,kf. (21204)

32 ((transcultur* or crosscultur* or multicultur*) adj5 (adapt* or translat* or modif* or tailor*)).ti,ab,kf. (533)

33 ((cultur* or ethnocultur* or transcultur* or crosscultur* or multicultur*) adj5 (divers* or competen* or appropriat* or responsive or relevan* or characteristic*)).ti,ab,kf. (38389)

34 ((cultur* or ethnocultur*) adj4 (specific* or sensitiv* or inclusiv*)).ti,ab,kf. (29812)

35 ((ethnic* or minority or minorities) adj5 (adapt* or translat* or modif* or tailor*)).ti,ab,kf. (1526)

36 ((ethnic or minority or minorities) adj2 (communit* or group*)).ti,ab,kf. (45850)

37 (CALD adj2 (communit* or group* or background*)).ti,ab,kf. (161)

38 ((cultur* or ethnocultur* or transcultur* or crosscultur* or multicultur*) adj2 linguist*).ti,ab,kf. (2995)

39 ((co-design* or co-creat* or codesign* or cocreat*) and (cultur* or ethnic* or ethnocultur* or transcultur* or crosscultur* or multicultur*)).ti,ab,kf,hw. (407)

40 ((participatory adj2 (design* or research or framework* or method*)) and (cultur* or ethnic* or ethnocultur* or transcultur* or crosscultur* or multicultur*)).ti,ab,kf,hw. (2672)

41 (action research and (cultur* or ethnic* or ethnocultur* or transcultur* or crosscultur* or multicultur*)).ti,ab,kf,hw. (755)

42 or/25-41 (200866)

43 24 and 42 (11303)

44 (patient* or consumer* or audience* or end user* or service user* or client* or public or parent* or mother* or father* or caregiver* or care giver*).ti,ab,kf,hw. (8730104)

45 43 and 44 (6725)

46 limit 45 to english language (6510)

**CINAHL (1936 -** July Week 2 2023**) via EBSCOhost**

**Date of search: July 16, 2023**

S1 (MH "Consumer Health Information") (13,301)

S2 TI ( ((consumer* or patient* or public) N1 "health information") ) OR AB ( ((consumer* or patient* or public) N1 "health information") ) (1,396)

S3 (MH "Health Promotion+") (74,038)

S4 TI ( health N1 (promotion* or campaign*) ) OR AB ( health N1 (promotion* or campaign*) ) (25,466)

S5 (MH "Communications Media") OR (MH "Information Resources") OR (MH "Posters") OR (MH "Pamphlets") OR (MH "Email") OR (MH "Instant Messaging") OR (MH "Text Messaging+") (108,113)

S6 TI "health communication" OR AB "health communication" (1,850)

S7 (MH "Patient Education+") (82,282)

S8 TI ( ((patient* or consumer*) N1 education*) ) OR AB ( ((patient* or consumer*) N1 education*) ) (16,924)

S9 (MH "Translational Medical Research") (83)

S10 TI ( ((knowledge N2 (translat* or transfer* or mobiliz* or mobilis* or exchange or implement* or disseminat* or uptake or adopt* or application* or apply or applie*)) and (service* or tool* or campaign* or program* or resource* or product*)) ) OR AB ( ((knowledge N2 (translat* or transfer* or mobiliz* or mobilis* or exchange or implement* or disseminat* or uptake or adopt* or application* or apply or applie*)) and (service* or tool* or campaign* or program* or resource* or product*)) ) (4,668)

S11 TI ( ((evidence N2 (translat* or transfer* or mobiliz* or mobilis* or exchange or implement* or disseminat* or uptake or adopt* or application* or apply or applie*)) and (service* or tool* or campaign* or program* or resource* or product*)) ) OR AB ( ((evidence N2 (translat* or transfer* or mobiliz* or mobilis* or exchange or implement* or disseminat* or uptake or adopt* or application* or apply or applie*)) and (service* or tool* or campaign* or program* or resource* or product*)) ) (5,150)

S12 TI ( ((research N2 (translat* or transfer* or mobiliz* or mobilis* or exchange or implement* or disseminat* or uptake or adopt* or application* or apply or applie*)) and (service* or tool* or campaign* or program* or resource* or product*)) ) OR AB ( ((research N2 (translat* or transfer* or mobiliz* or mobilis* or exchange or implement* or disseminat* or uptake or adopt* or application* or apply or applie*)) and (service* or tool* or campaign* or program* or resource* or product*)) ) (7,800)

S13 TI ( ((innovat* N2 (translat* or transfer* or mobiliz* or mobilis* or exchange or implement* or disseminat* or uptake or adopt* or application* or apply or applie*)) and (service* or tool* or campaign* or program* or resource* or product*)) ) OR AB ( ((innovat* N2 (translat* or transfer* or mobiliz* or mobilis* or exchange or implement* or disseminat* or uptake or adopt* or application* or apply or applie*)) and (service* or tool* or campaign* or program* or resource* or product*)) ) (1,531)

S14 TI ( (("best practice*" N2 (translat* or transfer* or mobiliz* or mobilis* or exchange or implement* or disseminat* or uptake or adopt* or application* or apply or applie*)) and (service* or tool* or campaign* or program* or resource* or product*)) ) OR AB ( (("best practice*" N2 (translat* or transfer* or mobiliz* or mobilis* or exchange or implement* or disseminat* or uptake or adopt* or application* or apply or applie*)) and (service* or tool* or campaign* or program* or resource* or product*)) ) (863)

S15 TI ( ("knowledge into practice" or "knowledge to practice" or "knowledge into action" or "knowledge to action") ) OR AB ( ("knowledge into practice" or "knowledge to practice" or "knowledge into action" or "knowledge to action") ) (746)

S16 TI ( ("evidence into practice" or "evidence to practice" or "evidence into action" or "evidence to action") ) OR AB ( ("evidence into practice" or "evidence to practice" or "evidence into action" or "evidence to action") ) (1,538)

S17 TI ( ("research into practice" or "research to practice" or "research into action" or "research to action") ) OR AB ( ("research into practice" or "research to practice" or "research into action" or "research to action") ) (2,248)

S18 TI ( ("innovation* into practice" or "innovation* to practice" or "innovation into action" or "innovation to action") ) OR AB ( ("innovation* into practice" or "innovation* to practice" or "innovation into action" or "innovation to action") ) (37)

S19 TI ( ("evidence based" N1 (program* or service* or intervention* or campaign* or tool* or resource*)) ) OR AB ( ("evidence based" N1 (program* or service* or intervention* or campaign* or tool* or resource*)) ) (7,719)

S20 TI ( (KT N1 (product* or tool* or intervention* or strateg* or campaign* or service*)) ) OR AB ( (KT N1 (product* or tool* or intervention* or strateg* or campaign* or service*)) ) (184)

S21 TI ( (brochure* or booklet* or pamphlet*) ) OR AB ( (brochure* or booklet* or pamphlet*) ) (5,035)

S22 TI ( ((app or apps or application*) N2 (mobile or device* or cell* or iphone* or android* or "smart phone*" or smartphone*)) ) OR AB ( ((app or apps or application*) N2 (mobile or device* or cell* or iphone* or android* or "smart phone*" or smartphone*)) ) (9,233)

S23 TI ( ("social media" or facebook or instagram or twitter) ) OR AB ( ("social media" or facebook or instagram or twitter) ) (18,294)

S24 S1 OR S2 OR S3 OR S4 OR S5 OR S6 OR S7 OR S8 OR S9 OR S10 OR S11 OR S12 OR S13 OR S14 OR S15 OR S16 OR S17 OR S18 OR S19 OR S20 OR S21 OR S22 OR S23 (334,846)

S25 (MH "Culture") OR (MH "Cultural Diversity") OR (MH "Cultural Safety") OR (MH "Cultural Values") (53,018)

S26 TI ( ((cultur* or ethnocultur*) N4 (adapt* or translat* or modif* or tailor*)) ) OR AB ( ((cultur* or ethnocultur*) N4 (adapt* or translat* or modif* or tailor*)) ) (7,008)

S27 TI ( ((transcultur* or crosscultur* or multicultur*) N4 (adapt* or translat* or modif* or tailor*)) ) OR AB ( ((transcultur* or crosscultur* or multicultur*) N4 (adapt* or translat* or modif* or tailor*)) ) (347)

S28 TI ( ((cultur* or ethnocultur* or transcultur* or crosscultur* or multicultur*) N4 (divers* or competen* or appropriat* or responsive or relevan* or characteristic*)) ) OR AB ( ((cultur* or ethnocultur* or transcultur* or crosscultur* or multicultur*) N4 (divers* or competen* or appropriat* or responsive or relevan* or characteristic*)) ) (20,661)

S29 TI ( ((cultur* or ethnocultur*) N3 (specific* or sensitiv* or inclusiv*)) ) OR AB ( ((cultur* or ethnocultur*) N3 (specific* or sensitiv* or inclusiv*)) ) (9,762)

S30 TI ( ((ethnic* or minority or minorities) N4 (adapt* or translat* or modif* or tailor*)) ) OR AB ( ((ethnic* or minority or minorities) N4 (adapt* or translat* or modif* or tailor*)) ) (765)

S31 TI ( ((ethnic or minority or minorities) N1 (communit* or group*)) ) OR AB ( ((ethnic or minority or minorities) N1 (communit* or group*)) ) (16,506)

S32 TI ( (CALD N1 (communit* or group* or background*)) ) OR AB ( (CALD N1 (communit* or group* or background*)) ) (150)

S33 TI (cultur* or ethnocultur* or transcultur* or crosscultur* or multicultur*) N1 linguistic* OR AB (cultur* or ethnocultur* or transcultur* or crosscultur* or multicultur*) N1 linguistic* (2152)

S34 TI ( (("co design*" or "co creat*" or codesign* or cocreat*) and (cultur* or ethnic* or ethnocultur* or transcultur* or crosscultur* or multicultur*)) ) OR AB ( (("co design*" or "co creat*" or codesign* or cocreat*) and (cultur* or ethnic* or ethnocultur* or transcultur* or crosscultur* or multicultur*)) ) (254)

S35 TI ( ((participatory N1 (design* or research or framework* or method*)) and (cultur* or ethnic* or ethnocultur* or transcultur* or crosscultur* or multicultur*)) ) OR AB ( ((participatory N1 (design* or research or framework* or method*)) and (cultur* or ethnic* or ethnocultur* or transcultur* or crosscultur* or multicultur*)) ) (1,048)

S36 S25 OR S26 OR S27 OR S28 OR S29 OR S30 OR S31 OR S32 OR S33 OR S34 OR S35 (92656)

S37 S24 AND S36 (8,474)

S38 TI ( (patient* or consumer* or audience* or "end user*" or "service user*" or client* or public or parent* or mother* or father* or caregiver* or "care giver*") ) OR AB ( (patient* or consumer* or audience* or "end user*" or "service user*" or client* or public or parent* or mother* or father* or caregiver* or "care giver*") ) OR MW ( (patient* or consumer* or audience* or "end user*" or "service user*" or client* or public or parent* or mother* or father* or caregiver* or "care giver*") ) (2,798,428)

S39 S37 AND S38 (4,837)

APA PsycInfo <1806 to July Week 2 2023>

**Date of search: July 16, 2023**

--------------------------------------------------------------------------------

1 health information/ (3332)

2 ((consumer* or patient* or public) adj2 health information).ti,ab. (483)

3 health promotion/ (28552)

4 (health adj2 (promotion* or campaign*)).ti,ab. (17610)

5 health communication.ti,ab. (2651)

6 client education/ (4605)

7 ((patient* or consumer*) adj2 education*).ti,ab. (5881)

8 knowledge transfer/ (3597)

9 ((knowledge adj3 (translat* or transfer* or mobiliz* or mobilis* or exchange or implement* or disseminat* or uptake or adopt* or application* or apply or applie*)) and (service* or tool* or campaign* or program* or resource* or product*)).ti,ab. (6661)

10 ((evidence adj3 (translat* or transfer* or mobiliz* or mobilis* or exchange or implement* or disseminat* or uptake or adopt* or application* or apply or applie*)) and (service* or tool* or campaign* or program* or resource* or product*)).ti,ab. (5065)

11 ((research adj3 (translat* or transfer* or mobiliz* or mobilis* or exchange or implement* or disseminat* or uptake or adopt* or application* or apply or applie*)) and (service* or tool* or campaign* or program* or resource* or product*)).ti,ab. (13244)

12 ((innovat* adj3 (translat* or transfer* or mobiliz* or mobilis* or exchange or implement* or disseminat* or uptake or adopt* or application* or apply or applie*)) and (service* or tool* or campaign* or program* or resource* or product*)).ti,ab. (2457)

13 ((best practice* adj3 (translat* or transfer* or mobiliz* or mobilis* or exchange or implement* or disseminat* or uptake or adopt* or application* or apply or applie*)) and (service* or tool* or campaign* or program* or resource* or product*)).ti,ab. (963)

14 ("knowledge into practice" or "knowledge to practice" or "knowledge into action" or "knowledge to action").ti,ab. (2993)

15 ("evidence into practice" or "evidence to practice" or "evidence into action" or "evidence to action").ti,ab. (9300)

16 ("research into practice" or "research to practice" or "research into action" or "research to action").ti,ab. (26680)

17 ("innovation* into practice" or "innovation* to practice" or "innovation into action" or "innovation to action").ti,ab. (226)

18 (evidence based adj2 (program* or service* or intervention* or campaign* or tool* or resource*)).ti,ab. (9518)

19 (KT adj2 (product* or tool* or intervention* or strateg* or campaign* or service*)).ti,ab. (60)

20 (brochure* or booklet* or pamphlet*).ti,ab. (5235)

21 mobile applications/ (2431)

22 ((app or apps or application*) adj3 (mobile or device* or cell* or iphone* or android* or smart phone* or smartphone*)).ti,ab. (6489)

23 exp social media/ (22647)

24 (social media or facebook or instagram or twitter).ti,ab. (25419)

25 or/1-24 (160435)

26 "Culture (Anthropological)"/ (22687)

27 cross cultural differences/ (55474)

28 cross cultural communication/ (2538)

29 cultural sensitivity/ (8266)

30 ((cultur* or ethnocultur*) adj5 (adapt* or translat* or modif* or tailor*)).ti,ab. (11209)

31 ((transcultur* or crosscultur* or multicultur*) adj5 (adapt* or translat* or modif* or tailor*)).ti,ab. (432)

32 ((cultur* or ethnocultur* or transcultur* or crosscultur* or multicultur*) adj5 (divers* or competen* or appropriat* or responsive or relevan* or characteristic*)).ti,ab. (44058)

33 ((cultur* or ethnocultur*) adj4 (specific* or sensitiv* or inclusiv*)).ti,ab. (20853)

34 Ethnic Identity/ (19059)

35 ((ethnic* or minority or minorities) adj5 (adapt* or translat* or modif* or tailor*)).ti,ab. (1112)

36 ((ethnic or minority or minorities) adj2 (communit* or group*)).ti,ab. (29884)

37 (CALD adj2 (communit* or group* or background*)).ti,ab. (116)

38 ((cultur* or ethnocultur* or transcultur* or crosscultur* or multicultur*) adj2 linguist*).ti,ab. (5815)

39 ((co-design* or co-creat* or codesign* or cocreat*) and (cultur* or ethnic* or ethnocultur* or transcultur* or crosscultur* or multicultur*)).ti,ab,hw. (813)

40 ((participatory adj2 (design* or research or framework* or method*)) and (cultur* or ethnic* or ethnocultur* or transcultur* or crosscultur* or multicultur*)).ti,ab,hw. (1987)

41 (action research and (cultur* or ethnic* or ethnocultur* or transcultur* or crosscultur* or multicultur*)).ti,ab,hw. (2113)

42 or/26-41 (177844)

43 25 and 42 (8289)

44 exp "racial and ethnic groups"/ (155651)

45 (patient* or consumer* or audience* or end user* or service user* or client* or public or parent* or mother* or father* or caregiver* or care giver*).ti,ab,hw. (1611404)

46 or/44-45 (1717045)

47 43 and 46 (4715)

48 limit 47 to english language (4606)

49 (202108* or 202109* or 202110* or 202111* or 202112* or 2022* or 2023*).up. (365455)

50 48 and 49 (610)
