# Supplementary material for: Exploring methods for creating or adapting knowledge mobilization products for culturally and linguistically diverse audiences: a scoping review
Source: Arch Public Health. 2024 Jul 22;82:111. doi: 10.1186/s13690-024-01334-0 (PMC11265177; doi:10.1186/s13690-024-01334-0)
Supplement: Supplementary file 4 — Supplementary Material 4. [file 13690_2024_1334_MOESM4_ESM.docx]

**Additional file 4: Characteristics of studies creating or adapting KMb products**

| **Study Characteristics** | | | **Population Characteristics** | | **KMb Product Characteristics** | | |
| --- | --- | --- | --- | --- | --- | --- | --- |
| **Author**  **Year**  **Country** | **Study design /**  **methods**  **Sample size** | **Objective and setting** | **Age, mean±SD (range)^*^**  **Males, n (%)** | **Primary language**  **Race**  **Condition** | **KMb product description and format** | | **Target end-users** |
| Abascal-Miguel  2022  Guatemala | - Quantitative - Pre–post intervention survey design - N=1572 | To evaluate the impact on vaccine uptake of the  social media campaign in Patzún, San Andrés Semetabaj, Solalá and Tecpán, Indigenous rural municipalities  in the Guatemala Central Highlands. | Age:  28 (22-39) (median, IQR)  Males:  574 (58%) | - Spanish, K’iche and Kaqchikel - Guatemalan - COVID-19 vaccine | Social media campaign on vaccine access including two animated video series with three short clips | | Public |
| Abbas-Dick  2018  Canada | - Qualitative - Participatory research with content analysis - N=11 | To work in partnership with Indigenous communities to create an eHealth breastfeeding resource for Indigenous families. | Age:  31 (18-57)  Males:  0 (0%) | - English - Indigenous - Breastfeeding | Online breastfeeding co-parenting resources including text, video, games, quizzes, and links to internet resources. | | Public (families) |
| Alexandrou  2021  Sweden | - Qualitative - Focus group and interviews - N=15 parents, N=15 nurses | To explore how the features and content of the MINISTOP 1.0 app could be refined to better support health behaviors in children, among both parents and nurses. | Age: Parents  34±5  Males:  1 (7%)  Age: Nurses  47**±**8  Males:  NR | - Parents: Somali, Arabic, Swedish - Healthy diets and activity promotion | Mobile health app that delivers a new comprehensive information on healthy diet and activity behaviors for  preschool-aged children every two weeks, over a period of six months. | | Parents |
| Ali  2019  UK | - Qualitative - Interviews - N=20 | To create a simple health literacy intervention aimed at supporting informed reproductive choice among members of UK communities practicing customary consanguineous marriage. | Age: NR  Males: NR | - Urdu - South Asian - Consanguineous marriage | Simple health literacy leaflet on reproductive choices. | | Public |
| Arnold  2011  USA | - Qualitative - Focus groups - N=13 | To describe how an American Indian community in Wyoming grew into an American Indian/Alaska Native women’s advisory committee, a culturally appropriate prenatal education booklet, and a national initiative for a non-profit organization. | Age: NR  Males: NR | - Various - Indigenous - Prenatal education | A comprehensive prenatal booklet that would “tell the story of the way to have a healthy pregnancy.” Presented in a conversational format. | | Parents |
| Avila  2023  USA | - Qualitative - Survey - NR | To linguistically and culturally adapt the Communicating the External Beam Radiotherapy Experience for Spanish speaking patients about radiation therapy. | Age: NR  Males: NR | - Spanish - Latinx in USA - Radiation therapy education | A graphic narrative discussion guide about radiation oncology treatments for Spanish patients in the USA. | | Patients |
| Baptista  2020  Portugal | - Qualitative - Interviews - N=15 | To translate and culturally adapt an English language decision aid addressing prostate cancer screening, so it can be used by Portuguese men. | Age:  61±4.9 (55-69)  Males:  15 (100%) | - Portuguese - White - Prostate cancer | Prostate cancer screening text-based decision aid. | | Patients |
| Best  2012  USA | - Mixed methods - Focus groups, interviews, RCT - N=200 | To work with a group of AA women to 1) identify important spiritual elements included in health communication materials, and create a spiritually-framed BCS message 2) evaluate effectiveness of spiritually-framed BCS message compared to a more traditional BCS message among AA women using a cognitive response analysis. | Age:  48.9±15.4 (25-88)  Males:  0 (0%) | - English - Black - BCS | Print and radio-based messaging for BCS information. | | Patients |
| Bilbrey  2018  USA | - Mixed methods - Focus groups, online surveys - NR | To create materials which help answer questions and support the decision-making process for families related to brain donation. | Age: NR  Males: NR | - Spanish - Latino - Brain donation | Bilingual brochure highlighting the benefits and process of brain donation. | | Patients (families) |
| Blazey  2023  USA | - Mixed methods - Surveys, semi- structured interviews - Phase 1: N=15 - Phase 3: N=10 | To create and evaluate the usability and acceptability of a prototype smartphone app for Black breast cancer survivors and their first-degree relatives to promote physical activity. | Phase 1  Age:  56.5±13.4  Males: 1 (7%)  Phase 3  Age:  45.9±15.7  Males: 0 (0%) | - English - AA - Cancer survivors | Smart phone app  to promote increased movement among  cancer survivors.  App included information, goal setting, notifications and prompts, motivational messaging, and knowledge components. | | Patients |
| Cabassa  2012  USA | Qualitative   - Meetings, email correspondence - N=NR   Quantitative   - RCT - N=150 | To describe the development of a depression *fotonovela* adapted for Latinos with limited English proficiency. | Age: NR  Males: NR | - Spanish - Latinx - Depression | Depression *fotonovela* adapted for Latinos with limited English proficiency. | | Patients |
| Caplan  2020  USA | - Qualitative - Interviews - N=62 | To adapt an evidence-based face-to-face Spanish language cognitive behavioral group depression course for use in a mobile app and to explore participants’ perceptions of the visual and audio content and the usability of the app in the Dominican population. | Age:  37 (18-64)  Males:  11 (18%) | - Spanish - Dominican - Depression | Mobile app to prevent depression and provide education about depression symptoms. | | Low-Income Patients |
| Celentano  2021  USA | - Qualitative - Focus groups - N=30   Evaluation   - Qualitative - Survey - N=134 | To create an HPV vaccine comic book for East African adolescents as a case study to illustrate such a process using the perspectives of parents and adolescents. | Mothers: Age:  41±5.6  Males: 0%  Youth  Age:  (14 – 17)  Age: 47% | - Somali, Amharic, and Tigrinya - East AA - HPV | Paper-based comic book for East AA adolescents about HPV vaccine. | | Patients |
| Chang  2021  Australia | - Mixed Methods - Surveys; focus groups - N=154 | Create the Mandarin version of the dementia and driving decision aid and measure its acceptability among Taiwanese people with dementia, family caregivers and practitioners. | 2017-Age:  36.8 ± 14.0  (19–68)  2018-Age:  36.3 ± 8.3 (25–52)  2019-Age:  41.6 ± 13.2 (23–68)  Males: NR | - Mandarin - Taiwanese - Dementia | Dementia and driving decision aid booklet. | | Patients and Caregivers |
| Crouse  2023  Australia | - Qualitative - Collaborative team setting - NR | To describe the scientific framework targeting cognitive, social, and emotional well-being used to create ‘Thrive by Five’ app. | Age: NR  Males: NR | - Various - Various - Early childhood development | ‘Thrive by Five’ app to be createed and implemented in 30 countries. Currently implemented in Afghanistan, Namibia, Kyrgyzstan, and  Uzbekistan | | Parents (of 0-5 years) |
| Cunningham-Erves  2022  USA | - Qualitative - Online survey and participant interviews - N=30 | To create a theory-based, culturally appropriate library of motivational messages for a social marketing campaign to promote COVID-19 vaccination among AA. | Age:  38.6±9.5  Males  6 (30%) | - English - AA - COVID-19 vaccination | Culturally appropriate, online, theory-based motivational messages targeting concerns around COVID-19 vaccines. | | Public (parents and adults) |
| Drago  2018  USA | - Qualitative - Interviews - N=22 | To characterize Latino parental perceptions of antenatal counseling in order to construct and validate a Spanish decision-aid to improve parental knowledge of prematurity after antenatal consults. | Age: NR  Males:  3 (14%) | - Spanish - Latino - Premature birth | Card-based English decision-aid for parents experiencing premature birth. | | Patients |
| Drenkard  2022  USA | - Qualitative - NR - NR | To create an internet-based program in Spanish and Portuguese to inform patients about lupus. | Age: NR  Males: NR | - Spanish and Portuguese - Latinx - Lupus | Hablemos de Lupus (Let Us Talk About Lupus) Internet-based program consisted of educational videos, self-management videos photos, and a website. | | Patients |
| Du Plessis  2022  South Africa | - Qualitative - Workshops and focus groups - N=324 | To create and  field-test proposed food based dietary guidelines for cultural appropriateness, consumer understanding,  acceptability, and feasibility among Tanzanian consumers. | Age:  35±11.6  Males:  0% | - Kiswahili and English - Tanzanian (Mainland and Zanzibar) - Healthy eating | Visual illustrations of 12 food based dietary guidelines tailored for various cultural groups in Tanzania. | | Public |
| Elliott  2022  Canada | - Qualitative - Survey and interviews - N=13 | To explore and understand the cultural and linguistic adaptation of a croup KT tool in Filipino communities. | Age:  (20 to ≥ 51)  Males:  4 (31%) | - Tagalog - Filipino - Croup | Online whiteboard animated video showing signs and symptoms of croup. | | Parents |
| Glennie  2022  Australia | - Qualitative - Crowdsourcing - Facebook metrics:   Users reached = 91,295  Times displayed = 638,294  ThurPlays = 75,591 | To present a novel, highly participatory pandemic prevention communication campaign that engaged individuals in remote Aboriginal  communities of the Northern Territory of Australia. | Age: NR  Males: NR | - Ndjébbana, Djambarrpuyngu, Kriol, Western Arrente, Anindilayka, English, Kunwinku - Aboriginal and Torres Strait Islander Australians - COVID-19 handwashing and distancing | 18 videos from native speaking Australians about pandemic prevention; videos distributed via  Facebook paid advertising to postal codes relevant to languages in videos. | | Public |
| Gordon  2015  USA | - Qualitative - Focus groups - N=76 | To describe the community engagement  processes and data collection efforts used to identify culturally  appropriate content and describe development of website content and design, and usability testing. | Age: NR  Males: NR | - Spanish - Latino - Kidney donation and transplantation | Website on routine transplant education. | | Patients |
| Grasaas  2019  Norway | - Qualitative - Interviews - N=11 | To describe the translation and cultural adaptation of the iCanCope with Pain mobile app into the Norwegian context and evaluate the app’s usability using a phased approach at the University of Agder laboratory. | Age:  (16-18)  Males: NR | - Norwegian - White - Adolescent pain | Mobile app containing resources for current best practices for pain self-management. | | Patients |
| Grinker  2015  USA | - Qualitative - Interviews - N=23 | To describe a methodology for translating outreach materials for ASD using the Autism Speaks First 100 Days Kit as an exemplar. | Age: NR  Males: NR | - Korean - East Asian - ASD | Autism Speaks First 100 Days Kit: text document. | | Patients (families) |
| Guttman  2013  Israel | - Qualitative - Interviews - N=104 | To create and present health rights information materials for a disadvantaged cultural minority, the Ethiopian immigrant community in Israel. | Age: NR  Males:  44 (42%) | - English - Middle Eastern/North African - Health rights information | Two styles of video: 1 video with a humorous storyline format, and 2 videos with a narrator relaying serious information. Two styles of print materials: a photonovella, and an illustrated booklet. | | Public |
| Hainsworth 2022  UK | - Qualitative - Online discussion panel - N=7 | To work in partnership with a group of men of African or African-Caribbean ancestry to plan and co-create a video intended to raise awareness about prostate cancer. | Age: NR  Males  7 (100%) | - AA-Caribbean - Prostate Cancer | A video featuring a success story of a man who had been diagnosed early with prostate cancer due to screening to raise awareness about prostate cancer risk in men and promote participation in a genetic screening study. | | Public |
| Hall  2022  Netherlands | - Quantitative - Questionnaire - N = 27 | To create a culturally appropriate and accessible health message to promote diabetic retinopathy screening and deliver a motivational message to promote acceptance of diabetic retinopathy screening irrespective of past eye health behavior in Kilimanjaro. | Age: NR  Males: NR | - Kiswahili - Tanzanian - Diabetic retinopathy | Create a culturally relevant comic strip to assist with barriers faced by people with diabetes. | | Patients |
| Hamdiui  2021  Netherlands | - Mixed-methods - Focus groups; survey; RCT - N=44 - N=482 - N=1564 | To create a short culturally sensitive educational video to facilitate informed decision‐making for cervical cancer screening among Turkish‐ and Moroccan‐Dutch women. | Age:  (30-60)  Males:  0 (0%) | - Turkish, Moroccan-Arabic, and Moroccan-Berber - Moroccan and Turkish - Cervical Cancer | Three short videos for informed decision making around cervical cancer screening. | | Public |
| Harvey  2011  USA | - Qualitative - Interviews - N=20 | To create, produce and disseminate culturally and linguistically appropriate health brochures in community health center serving populations of low health literacy, in Philadelphia. | Age:  (20-60)  Males:  NR | - Spanish - Latinx - Health information topics | Written health brochures on common health information to promote health literacy. | | Public |
| Hashim  2013  UAE | - Mixed methods - Focus groups, survey - N=17 | To investigate the content and design preferences of printed health education leaflets among Arabic communities, conducted in clinic waiting areas at public hospital. | Age:  47.4±14.3 (17-70)  Males:  9 (53%) | - Arabic - Middle Eastern/North African - Preferences for health education materials | Diabetes health education materials in a trifold brochure. | | Public |
| Hempler  2015  Denmark | - Qualitative - Design-based research approach - N=18 | To create culturally sensitive dialog tools to support person-centered dietary education targeting Pakistani immigrants in Denmark with type 2 diabetes. | Age: NR  Males: NR | - English - South Asian - Type 2 diabetes | Diabetes dialog tools including interactive engagement, online videos, and text. | | Patients |
| Hodge  2012  USA | - Qualitative - Focus group - N=132 | To create a cancer symptom management toolkit for American Indian cancer survivors | Age: NR  Males:  37 (28%) | - English - American Indian - Cancer | | ‘Weaving Balance into Life’ cancer symptom management toolkit consisted of self-management guide, resource directory, and motivational video | Public |
| Hong  2022  USA | - Qualitative - Interviews - N=17 | To create a culturally tailored mHealth program called Wellness Enhancement for Caregivers (WECARE) to improve caregiving skills, reduce distress, and improve the psychosocial well-being of Chinese American family caregivers of persons with dementia. | Age: NR  Males:  1 (20%) | - Chinese - Chinese - Dementia | mHealth WeChat-based program containing articles with audio recordings and short videos, and s “private chat,” “group chat,” and “video chats” | | Public |
| Jameel  2023  Australia | - Qualitative - Co-design consultations; online workshops - NR | To detail the available literature in ED tools for First Nations and create a pre-ED communication tool to support concerns of Indigenous patients in a metro-urban ED in Melbourne. | Age: NR  Males: NR | - English - Indigenous - ED care | Comic strip communication tools for providing information on ED care. | | Patients |
| Jiang  2021  USA | - Mixed Methods - Focus groups; Interviews; RCT; Survey - N=58 (focus groups) - N=40 (pilot study) - N=100 (RCT) | To assess the feasibility, acceptability, and preliminary efficacy of a fully automated bidirectional SMS cessation intervention adapted for Vietnamese smokers. | Age:  38.9±8.2 (21-55)  Males:  98 (98%) | - Vietnamese - Vietnamese - Smoking | SMS text messages designed to increase knowledge about smoking, motivation to quit, and strategies to help quit. | | Public |
| Jiang  2022  USA | - Qualitative - Semi-structured Interviews - N=20 | To examine the feasibility, acceptability, and preliminary effectiveness of a culturally adapted, linguistically appropriate WeChat-based mobile messaging smoking cessation intervention among Chinese immigrant smokers. | Age:  (24-62)  Males:  13 (65%) | - Chinese - Chinese - Smoking | Text messages sent through WeChat, including educational messages, self-efficacy messages, and skill messages. | | Public |
| Kandasamy  2022  Canada | - Quantitative - Surveys - N=30 | To create a series of evidence-based narrative messages with engaging videos to address key sources of vaccine hesitancy/lack of confidence among South Asians living in Canada and collaborate with youth ambassadors to disseminate the videos and foster evidence-based dialogue. | Age:  23.3 (18-29)  Males:  6 (20%) | - English; Punjabi; Hindi; Tamil; Gujarati - South Asian - COVID-19 vaccine | Culturally sensitive videos about the COVID-19 vaccine. | | Public |
| Kayler  2023  USA | - Qualitative - Meetings; Cognitive interviews; Focus groups   Development   - N=116   Refinement   - N=31 | Develop (402a) and refine (402) of an educational series of animated videos “Living Donation and Kidney Transplantation Information Made Easy” (KidneyTIME) | Development  Age:  51±14  Males:  47 (41%)  Refinement  Age:  51 (26-75)  Males:  16 (52%) | - English - African and Latinx American - Living kidney donation and transplant | 12 animations focused on living kidney donation, with target audience members participating in the refinement process were predominantly AA and white | | Patients and families |
| Kerr  2021  USA | - Qualitative - Focus groups - N=63 | To create a multifaceted community-informed PrEP-focused HIV prevention messaging campaign for AA young adults in Louisville, KY. | Age:  (18-29)  Males: NR | - English - AA - HIV PrEP | A multimedia (print, digital, Internet radio, website, social media) campaign to increase PrEP awareness among AA young adults. | | Public |
| Ko  2014  USA | - Qualitative - Focus groups; meetings - Patient focus groups, N=30 | To create a Spanish language version of an evidence-based multimedia colorectal cancer screening decision aid | Patient focus groups:  Age: 56±4.5  Males:  14 (47%) | - Spanish - Latino - Colorectal cancer | | Colorectal cancer multimedia decision aid including a video with narration, images and animations | Public |
| LaMonica  2022  Australia | - Qualitative - Workshops - N=174 parents and caregivers | To create and iteratively refine a novel mHealth  app for parents and facilitate the successful implementation and  adoption of Thrive by Five to promote and optimize socio-emotional and cognitive treatment of children from  birth to age 5 years. | Age: NR  Males: NR | - Countries in Africa, Central Asia, Southeast Asia, South Asia, Middle East, North and South America - Various - socio-emotional and cognitive treatment of children | ‘Thrive by Five’ app to be created and implemented in 30 countries. | | Parents |
| Lee  2019  USA | - Qualitative - Focus groups - N=20 | To demonstrate how a culturally targeted and tailored mobile screening (mScreening) was created to promote the uptake of Papanicolaou tests and HPV among young Korean American immigrant women. | Age:  26 (21-29)  Males:  0 (0%) | - Korean - East Asian - Cervical cancer | HPV mScreening mobile app. | | Public |
| Leiter  2023  USA | - Qualitative - Interviews - N=10 | Adapt a multimedia chemotherapy educational intervention to meet the unique needs of Spanish and English-speaking Latinos. | Age: NR  Males:  8 (80%) | - Spanish; English - Latinx - Chemotherapy | A multimedia parent educational intervention containing five videos and companion booklets. | | Patients |
| LeLaurin  2022  USA | - Qualitative - Interviews; focus groups - N=47 | To provide comprehensive resources for stroke caregivers by creating the English- and Spanish-language Resources & Education for Stroke Caregivers’ Understanding & Empowerment (RESCUE) websites. | Age:  58.5±11.3  Males:  2 (4%) | - Spanish - Latinx - Stroke | RESCUE website consisted of fact sheets, a list of resources, and self-management tools, for those acting as caregivers for stroke patients. | | Patients (their care givers) |
| Lemon  2022  Australia | - Qualitative - Yarning sessions - N=35 | To create a health promotion campaign to prevent prenatal alcohol exposure in Alice Springs, NT. | Age: (≥18)  Males: NR | - English - Aboriginal &Torres Strait Islander - Prenatal alcohol exposure; Fetal alcohol syndrome | Television commercials and radio advertisements portraying a life course journey from planning a pregnancy, to conception, birth and create a healthy young man. | | Public |
| Li  2012  USA | - Qualitative - Interviews - N=5 | To create, pilot test and evaluate an innovative, culturally relevant CD-ROM with a focus on hypertension education and management, directed at the older Chinese immigrant population, at Asian health clinics in San Francisco. | Age:  74.3±4.8 (71-81)  Males:  4 (20%) | - Mandarin - East Asian - Hypertension | Hypertension CD-ROM including visual aids and audio features. | | Patients |
| Liu  2021  China/Canada | - Qualitative - Meetings and semi-structured interviews - Evaluation by experts (N=23) and patients (N=39) | To translate and culturally adapt Cardiac College^TM^ educational materials for Mandarin-speaking people living with CVD in China (domestic version) and internationally. | Age:  58.4±11.2  Males:  NR (61.5%) | - Mandarin and English - Chinese - Cardiovascular disease | Cardiac College^TM^ is an online tool that aims to help people understand their heart disease and its’ treatment, and take control of their health; consisting of booklets, slide presentation and a website. | | Patients |
| Maertens  2017  USA | - Qualitative - Focus groups - N=47   Evaluation   - Quantitative - RCT - N=1294 | To investigate how to modify a previously created web-based intervention that provided individually tailored information about HPV to improve its use among the Latino population. | Age: ≥18  Males:  3 (6%) | - Spanish - Latinx - HPV vaccine | Combatting HPV Infection & Cancer (CHiCOS) a culturally tailored educational website featuring messages tailored to address concerns about HPV vaccination. | | Parents and Youth |
| Malamsha  2021  Tanzania | Treatment   - Mixed Methods - Survey; Focus groups - N=111 (Survey) - N=24 (focus groups)   Validation   - Mixed Methods - Survey; Interviews; Observation - N=32 | To create a child sexual abuse prevention education mobile learning game to target the 3 to 5-year-old group, tailored for Tanzania as a case study. | Phase 1  Age:  (20-41 and above)  Males:  59 (44%)  Phase 2  Age:  (20-41 and above)  Males:  14 (44%) | - Swahili - Tanzanian - Child sexual abuse | A user-friendly children’s game app for prevention of sexual abuse. | | Children |
| Martinez  2023  USA | - Quantitative - Surveys - N=6 | To create and evaluate pragmatic recommendations for culturally and comprehensibly enhanced behavior intervention documents for parents of children with ASD. | Age: NR  Males:  0 (0%) | - English - AA/Black; Caucasian American/White - ASD | Standard packets and enhanced pamphlets describing training for behavioral techniques. | | Parents |
| Materia  2020  USA | - Qualitative - Face-to-face sessions - NR | To report on the treatment of the Diabetes Prevention Program – South Africa SMS system, specifically, the evidence-informed decision-making processes that researchers went through to design and implement the SMS system to support intervention reach and support participants and community health workers. | Age: NR  Males: NR | - Xhosa - South African - Diabetes | SMS text messages used alongside a Diabetes Prevention Program to encourage healthy behavioral habits. | | Public |
| Mathieson  2012  New Zealand | - Qualitative - Interviews - N=16 | To adapt an existing cognitive behavioural therapy–based, guided self-management intervention for near-threshold MH syndromes in primary care, for Maori, and to examine its acceptability and effectiveness. | Age:  38.9 (20-65)  Males:  9 (19%) | - te reo (Maori language) - Indigenous - MH syndromes | Self-management booklets on relevant MH issues. | | Patients |
| Mauka  2021  Tanzania | - Qualitative - Small group discussions; Focus groups - N=15 (discussions) - N=20 (Focus groups) | To present the development process of an mHealth app (Jichunge app) for PrEP adherence among men who have sex with men and female sex workers in Tanzania. | Discussions  Age:  26 (NR)  Males: NR  Focus Groups  Age:  27 (NR)  Males: NR | - English; Swahili - African - HIV Prevention | mHealth app to promote adherence to PrEP including educational documents and pictorial presentations. | | Public |
| McFarlane  2019  USA | - Qualitative - Focus groups - N=20 | To evaluate the utility of a suite of clinical trial multimedia tools (website, decision aid, and animations) which will be utilized at a cancer center in a metropolitan city with a predominantly Hispanic population. | Age: NR  Males:  5 (25%) | - English; Spanish - Hispanic - Cancer | A website, four animations, and a decision aid. | | Patients |
| Meherali  2021  Canada | - Mixed methods - Focus groups, surveys - N=68 | To (1) create Acute Otitis Media KT tools for Canadian parents, (2) adapt the KT tools across cultural contexts, and (3) evaluate the usability of the adapted KT tools. | Age: (0-50)  Males:  4 (6%) | - Urdu - South Asian - Acute otitis media | Digital arts-based KT tools on how to manage acute otitis media. | | Parents |
| Montague Lecturer  2022  UK | - Qualitative - Semi-structured Interviews - N=30 | To evaluate the feasibility of using the digital animation to increase physical activity and exercise levels in asylum seeking communities. | Age: (20-54)  Males:  13 (43%) | - Middle Eastern - Physical activity | Short 1-minute animation to encourage and educate everyday activities to increase physical activity levels. | | Public |
| Norris  2021  USA | - Qualitative - Focus groups - N=25 | To create a multicultural adaptation of the Mighty Girls program using a mobile app that is less costly to disseminate and is acceptable to parents of 7^th^ grade girls. | Age: (10-14)  Males:  0 (0%) | - English - Latinx - Sexual health | A narrative-generating app to support sexual health behavior change. | | Patients (families) |
| Pathak  2021  USA | - Mixed Methods - Card sorting; semi-structured interviews - N=10 | To create text messages for use within an adaptive smartphone app for low-income ethnic minority patients with diabetes diagnosis and depression symptoms. | Age: NR  Males: NR | - English; Spanish - Latinx - Diabetes and depression | Text messages within app–based texting intervention that seeks to encourage physical activity in low-income minority patients with diabetes diagnoses and depression symptoms. | | Patients |
| Payan  2020  USA | - Quantitative - RCT - N=240 | To assess the efficacy of a Spanish educational brochure and a community health worker intervention about breast cancer compared to a control group. | Age:  52.3±8.8 (35-72)  Males:  0 (0%) | - Spanish - Latina - Breast Cancer | Brochure containing information about the advantages and disadvantages of preventions strategies for breast cancer and address common misconceptions. | | Patients |
| Povey  2022  Australia | - Qualitative - Surveys, interviews, meetings, workshops - N=75 | To present an in-depth account of the second phase of participatory design in creating the Aboriginal and Islander Mental Health Initiative for Youth (AIMhi-Y) app. | Age:  15.1±1.7  (8-18)  Males:  33 (44%) | - English and Tiwi - Aboriginal or Torres Strait Islander - MH | Smartphone-based app that integrates culturally adapted low-intensity cognitive behavioral therapy, psychoeducation, and mindfulness-based activities into  a universal early intervention; presented through relatable storytelling and appealing user interface. | | Patients |
| Quintana  2022  Argentina | - Qualitative - Interviews and social media - N=40 | To design and assess bilingual audiovisual materials by two social networks, for patients with RA in the *qom* community in Argentina. | Age:  45±11.5  Males:  12 (30%) | - Spanish - Latinx - RA | Video animations about RA distributed via Facebook and WhatApp. | | Patients |
| Rami  2018  Egypt | - Quantitative - RCT - N=20 | To translate and culturally adapt the Behavioral Family Psycho-Education Program and conduct a preliminary efficacy evaluation for outpatients suffering from schizophrenia. | Age:  (18-65)  Males: NR | - Arabic - Middle Eastern/North African - Schizophrenia | Informational leaflets about schizophrenia, high emotionally expressive families, and training materials for communication and problem-solving skills. | | Patients (families) |
| Santos  2021  Brazil | - Qualitative - Situational diagnosis - N=15 children - N=11 expert judges for validation | To build and validate  the content of booklet “It’s time to get my vein: what do I do?”, together with expert judges in pediatrics, for preparation of children in need of peripheral intravenous catheterization. | Age:  38.9±11.1  Males:  11 (100%) | - NR - Brazilin - Peripheral intravenous catheterization | Booklets administered to families and children prior to peripheral intravenous catheterization. | | Families (children) |
| Sharpe  2013  USA | - Qualitative - Interviews - N=32 | To describe the process and application of formative assessment with women attending an American Indian women’s clinic to create and pretest a culturally tailored brochure. | Age:  41 (20-61)  Males:  0 (0%) | - English - Indigenous - Women with HPV | Informational brochure with HPV vaccine recommendations, its availability, and accessibility. | | Patients |
| Songtaweesin  2021  Thailand | - Qualitative - Focus groups with Young Men who Sleep with Men (YMSM); N=23 - Interviews with key informants/ providers; N=15 | To adapt the P3 (Prepared, Protected, emPowered) app, to improve PrEP adherence and persistence for YMSM in Thailand. | Users  Age:  20 (18-21)  Males:  100%  Providers  Age:  40 (26-60)  Males: NR | - Thai - Taiwanese - Prophylaxis use | Mobile application that uses social networking and game-based elements along with evidence-based features to promote behavioral change of YMSM on PrEP use. | | Patients |
| Stanley  2018  USA | - Qualitative - Focus groups - N=24 | To describe formative research undertaken to guide adaptation for AI youth of a prevention intervention, Be Under Your Own Influence (BUYOI), previously found to be effective in reducing substance use among middle-school youth. | Age: NR  Males: NR | - English - Indigenous - Substance use | BUYOI paper-based messages, targeted to middle-school youth. | | Public |
| Teles  2021  Portugal | - Qualitative - Meetings and consultation - N=4 | To create a culturally adapted version of the World Health Organization iSupport dementia program for informal caregivers to the Portuguese culture. | Age: NR  Males: NR | - Portuguese - Portuguese - Dementia | iSupport, an online self-help program designed to provide education, skills training, and social support to informal caregivers of people with dementia. | | Caregivers |
| Tolentino  2022  USA | - Qualitative - Interviews - NR | To describe components of a social media initiative (Next Gen Hawai’i) on COVID-19 messaging to engage Native Hawaiian, Pacific Islander and Filipino youth. | Age: NR  Males: NR | - Chuukese, Chamorro, Marshallese, Samoan, Hawaiian, Ilocano, and Tagalog - Hawaiian, Pacific Islander and Filipino - COVID-19 | Videos and infographics of COVID-19 relevant public health topics for youth. | | Public (youth) |
| Umaefulam  2022  Canada | - Qualitative - Semi-structured interviews - Cohort 1, N=7 - Cohort 2, N=9 | To adapt the Early RA decision aid for use with Canadian Indigenous patients. | Cohort 1  Age: (37-61)  Males:  7 (100%)  Cohort 2  Age: (28–69)  Males:  9 (100%) | - English - Indigenous - RA | Two-page decision aid describing symptoms, therapies and their side effects about RA (Print format). | | Patients |
| Valenzuela-Araujo  2021  USA | - Qualitative - Survey - 79 parent/child dyad | To evaluate a Spanish-language educational  video to provide immigrant Latino families with information  about the USA healthcare system and explore parents’ views on video content, style, length, and cultural appropriateness. | Age:  30.1±6.1  Males:  NR | - Spanish - Latinx - Navigating healthcare information specifically for fever in infants | Nine-minute educational video supporting healthcare navigation and  engagement skills of Spanish-speaking Latino parents of infants. | | Parents and Families |
| van der Steen  2013  Canada | - Qualitative - Content analysis of transcribed materials - NR | To create a decision-making booklet on palliative care issues. To understand which aspects are sensitive from an ethical and cultural point of view within the respective cultural contexts in giving shape to palliative care in dementia. | Age: NR  Males: NR | - Dutch, Japanese and Italian - White/East Asian - Dementia | A booklet on comfort care at the end of life for people with dementia. | | Patients (families) |
| Van Son  2014  USA | - Qualitative - Interviews - N=10 | To describe a project in which culturally targeted diabetes education materials for older Russian-speaking immigrants were designed and created, delivered at diabetes clinics. | Age:  (over 65)  Males: NR | - Russian - White - Diabetes | 12 educational documents created with Russian on one side and English on the other to support patients make better health decision with regards to food. | | Patients |
| Versteegh  2022  Australia | - Qualitative - Interviews, meetings - N=80 | To create a First Nations-specific, multi-lingual, digital Asthma APP, based on current pediatric pictorial asthma flipchart. | Children  Age:  Median (IQR) 6.5 (3.9-11.8)  Males:  52 (65%)  Carers  Age:  >15 years  Males: NR | - English, Tiwi, Murrinh-Patha, Yolngu Matha, Kriol, Warlpiri, Central Arrernte, and Pitjantjatjara - First Nations - Asthma | Multi-lingual Asthma app, with a “voice-over” in seven local First Nations languages and English, using a mixture of static and interactive formats. | | Parents |
| Wall  2022  USA | - Qualitative - Interviews - N=77 | To create and assess whether generic versus  culturally targeted or personally tailored educational videos,  produced for Black men in Black owned barber shops, differentially affect organ donor registration after viewing. | Age:  Median 36  Males:  100% | - English - Black, AA, or Afro-Caribbean/ West Indies heritage - Organ donation | 4-6-minute educational videos representing a generic scenario and tailored scenario for Black men. | | Patients |
| Wright  2023  Canada | - Qualitative - Recounting of stories - N=5 | A community-engaged KT initiative was created at suggestion of Indigenous mothers  participating in a research study examining experiences using health care to meet the health needs of their infants. | Age: NR  Males: 0% | - English - Indigenous - Healthcare experiences of Indigenous mothers with relation to their children | Healing the Hurt video series and website educational resource to share Indigenous mothers' messages with healthcare providers. | | Parents |
| Wu  2021  Australia | - Qualitative - Focus groups and semi-structured interviews   Round 1   - N=29   Round 2   - N=5 | To create and test acceptability of a  self-management intervention (WeCope) in terms of its scope, social and cultural relevance and sensitivity for Chinese-Australian immigrants affected by cancer. | Round 1  Age:  59.5±11.3  Males:  6 (21%)  Round 2  Age: NR  Males: NR | - Cantonese and Mandarin - Asian Australian - Cancer | Six A5-sized booklets addressing various coping skills regarding cancer. | | Patients |
| Yeager  2022  USA | - Qualitative - Pre-post survey assessment - N=50 | To create and test the feasibility and preliminary efficacy of videos about participation in cancer clinical trials, with one video culturally tailored to Black patients. | Age:  53±15  Males:  0% | - English - Black - Breast cancer; participation in clinical trials | Two 7-min videos focusing on breast cancer patients describing experiences participating in clinical trials, supplemented with doctors and research staff explaining key research concepts. | | Patients |
| Zerafa  2022  Australia | - Qualitative - Co-design workshops - N=49 | To create an inclusive resource about incontinence to reach a diverse and broad population, independent of written or spoken language | Age: NR  Males: NR | - Independent of written or spoken language - Incontinence | Prototypes created and converted into animated videos, showcasing three types of incontinence: urgency, stress and frequency. | | Patients |

Abbreviations: African American = AA; Autism Spectrum Disorder = ASD; Breast Cancer Screening = BCS; CALD= Culturally and Linguistically Diverse; Coronavirus = COVID; Emergency Department = ED; Human Immunodeficiency Virus = HIV; Human Papilloma Virus = HPV; Knowledge Mobilization = KMb; Knowledge Translation = KT; Mental Health = MH; Not Reported = NR; Pre-Exposure Prophylaxis = PrEP; Randomized Controlled Trial = RCT; Rheumatoid Arthritis = RA; Short Message Service = SMS; United Kingdom = UK; United States of America = USA

* age in years
