# Supplementary material for: Exploring methods for creating or adapting knowledge mobilization products for culturally and linguistically diverse audiences: a scoping review
Source: Arch Public Health. 2024 Jul 22;82:111. doi: 10.1186/s13690-024-01334-0 (PMC11265177; doi:10.1186/s13690-024-01334-0)
Supplement: Supplementary file 5 — Supplementary Material 5. [file 13690_2024_1334_MOESM5_ESM.docx]

**Additional file 5: Processes of creating or adapting KMb products**

| **Study** | **KMb Product Creation/Adaptation Process** |
| --- | --- |
| Abascal-Miguel  2022  Guatemala | Product creation: development of a new KMb product (culturally tailored social media campaign on COVID-19 vaccine uptake among indigenous populations in Guatemala)  Literature/product search: NR  Stakeholder engagement: a qualitative human-centred design approach through in-person, in-depth key informant interviews and community focus groups to understand better vaccination barriers, including access, supply, trust and fear  Resources utilized: trained multilingual community members, narration is provided by local voice talent  Evaluation: survey-based evaluation through social media |
| Abbas-Dick  2018  Canada | Product creation: development of a new KMb product (eHealth resource for Indigenous families created)  Literature/product search: compiled generic resources on breastfeeding  Stakeholder engagement: Indigenous mothers and committee members reviewed generic resources and provided feedback for culturally relevant resources; interviews used to gather feedback from mothers about new resource  Resources utilized: committee members and health care professionals (health care providers were involved in providing breastfeeding education/support), lactation consultant, education specialist, website developer, graphic designer, animator  Evaluation: Unstructured discussions with Indigenous mothers from development phase, advisory members, additional mothers recruited for resource feedback on cultural suitability |
| Alexandrou  2021  Sweden | Product creation: adaptation of existing KMb product (MINDSTOP mobile health application for parents to promote healthy eating and physical activity among Somali, Arabic and Swedish-speaking parents)  Literature/product search: NR  Stakeholder engagement: focus groups with nurses and Somali, Arabic and Swedish-speaking parents; collaborated with the Swedish National Dental Health Agency to create a comprehensive theme on dental health for preschool-aged children  Resources utilized: translator; nurses; external transcribing firm; occupational therapist  Evaluation: MINDSTOP 2.0 is currently undergoing evaluation |
| Ali  2019  UK | Product creation: development of a new KMb product (health literacy leaflet for Pakistani or British Pakistani)  Literature/product search: preparatory work involved developing networks with community organizations  Stakeholder engagement: semi-structured interviews, focus groups and workshops with men and women from community to understand the context of cousin marriage and extend insight into attitudes, knowledge and information needs; four participatory workshops (one mixed sex and three single sex) attended by 8–14 participants with some participants carrying on from the first round; group exploration of alternative potential content and formats for resource vignette-led community level group discussions on wider social context; prototype leaflet development; fine-grained piloting and refinement; local rollout: printing and companion audio/video clips  Resources utilized: multi-lingual nurse researcher, public health researcher, two designers with expertise in participatory design, an anthropologist, a product designer and a multilingual research assistant, community organizations and leaders  Evaluation: NR |
| Arnold  2011  USA | Product creation: development of a new KMb product (booklet using the concept of the medicine wheel for American Indian/Alaska Native Families)  Literature/product search: NR  Stakeholder engagement: investigators started by building a trusting relationship with American Indian women from reservations and those attending health conferences; approached them to join volunteer committee; committee consisted of 13 volunteers, staff and volunteer faculty members; women were encouraged to conduct focus groups on their respective reservations with other moms and health care providers  Resources utilized: American Indian/Alaska Native graphic artist  Evaluation: survey distributed with booklet in clinics |
| Avila  2023  USA | Product creation: adaptation of existing KMb product (clinical decision guides from English to Spanish)  Literature/product search: Communicating the External Beam Radiotherapy Experience (CEBRE) clinical discussion guide series  Stakeholder engagement: involvement of interprofessional team consisting of local and Spanish speaking clinicians and educators, this group participated in iterative reviews and resource revisions  Resources utilized: professional healthcare translator aligned with a USA Spanish speaking audience; interprofessional and multilingual team including radiation oncologist, emergency physician, medical Spanish educator, information designer, medical student; information designer  Evaluation: understandability and actionability were assessed through the Patient Education Materials Assessment Tool (PEMAT) |
| Baptista  2020  Portugal | Product creation: adaptation of existing KMb product (decision aid for prostate cancer screening for Portuguese men)  Literature/product search: searched for prostate cancer screening decision aids from the Ottawa Hospital Research Institute; applicable decision aids were critically appraised; the ‘Making the Best Choice’ decision aid was selected because it was presented in two different formats; several years have lapsed since the original decision aid was developed. Therefore, a rapid review of clinical practice guidelines and systematic reviews of randomized controlled trials was performed to ensure the data provided were up-to-date  Stakeholder engagement: process coordinator and linguistic expert engaged to ensure that culturally and technically inappropriate recommendations were removed from decision aid; professional translator, native speaker of English, fluent in Portuguese; after comprehension testing with 15 Portuguese men who were interviewed to share their impressions, proofreading was conducted by two native Portuguese speakers selected by the process coordinator, who had not read the decision aid before  Resources utilized: translation committee (process coordinator; linguistic expert; translators)  Evaluation: NR |
| Best  2012  USA | Product creation: development of a new KMb product (spiritually-framed BCS messages among African American Women)  Literature/product search: NR  Stakeholder engagement: three nominal group sessions with African American women were conducted to identify and prioritize the most important spiritual elements to be included in BCS messaging  Resources utilized: NR  Evaluation: RCT to evaluate the effectiveness of the spiritually-framed messaging compared to more traditional messaging for BCS |
| Bilbrey  2018  USA | Product creation: development of a new KMb product (culturally appropriate brain donation decision aid materials for Latino community members)  Literature/product search: gathered information about existing rates of brain donation from successful Alzheimer’s Disease Centers to identify key barriers to brain donation; review of literature  Stakeholder engagement: two local focus groups in Spanish with promotoras (indigenous persons who go into homes and provide health education and basic health screenings to Latino families) to ask about their attitudes and beliefs toward brain donation; survey in English and Spanish that was emailed to local Latino health providers and Latino community members about current topic knowledge; multi-cultural, multi-lingual working group decided that a brochure to take home for undecided enrollees would be beneficial to discuss brain donation with family members; feedback was requested from local promotoras for ease of conceptual understanding, review of graphics, and approachability of material; a near final version was then translated by bilingual staff  Resources utilized: NR  Evaluation: NR |
| Blazey  2023  USA | Product creation: development of a new KMb product (culturally appropriate movement APP for cancer survivors among Black and their first-degree relatives)  Literature/product search: literature and theory informed the smartphone APP (MoveTogether)  Stakeholder engagement: community members/leaders participated in interviews to identify needs, context, and perspectives in creating the APP  Resources utilized: community leaders (self-identified as Black and led community organizations)  Evaluation: working prototype usability tested by community members, consistent with user-centered design methods, modifications to the APP were made after every 1 or 2 testing sessions; refined APP was evaluated in a 5-week, single-arm pilot study |
| Cabassa  2012  USA | Product creation: development of a new KMb product (*fotonovela* for depression for Latinos with limited English proficiency)  Literature/product search: described findings from two studies that informed the content and story line development of our *fotonovela*  Stakeholder engagement: formulation of objectives at team meetings; storyline development through iterative process that stakeholders reviewed and provided feedback; community members, clinicians, and professional writers reviewed the script and provided suggestions for revisions; attended script reading  Resources utilized: pharmacist, social work researcher, graphic artist, *fotonovela* producer to lead the production process; multi-stakeholder input (e.g., community members, clinicians, professional writers) to inform the development of our *fotonovela*; for filming of *fotonovela*: actors, project director, producer and photographer, hair/makeup artist  Evaluation: RCT to test a novel culturally-adapted patient education intervention to increase engagement of Hispanics in depression treatment |
| Caplan  2020  USA | Product creation: adaptation of existing KMb product (mobile APP for depression among low-income primary care patients in the Dominican Republic)  Literature/product search: review of the literature of previous MH apps and methods of cultural adaptation; theoretical literature on principles of APP design; implementation research conducted in low resource settings  Stakeholder engagement: patients and staff in a Primary Attention Unit in Santo Domingo assessed acceptability and cultural relevance of the content through interviews; patients from clinics in Boca Chica and Santo Domingo provided feedback on the overall acceptability of the APP in terms of content and usage  Resources utilized: animator; an interdisciplinary team composed of psychologists, nurses, instructional designers and public health professionals  Evaluation: feedback about the APP content and sociocultural appropriateness were provided by clinical staff |
| Celentano  2021  USA | Product creation: development of a new KMb product (HPV vaccine comic book for East African adolescents)  Literature/product search: review of the literature on perceptions of HPV vaccine and uptake among East African mothers and their adolescent children  Stakeholder engagement: focus groups with East African mothers (with vaccinated and unvaccinated children) in King County, Washington to inform development; to understand mothers’ socio-cultural beliefs and information needs about the HPV vaccine and to gather input on a comic book mockup developed by the research team  Resources utilized: East African mothers, community members  Evaluation: mothers and their adolescent children were recruited to participate in ethnolinguistic-centric educational dinners on HPV vaccination (Somali or Amharic only); each participating child was given a copy of the comic book and asked to respond to four open-ended questions to access for acceptability |
| Chang  2021  Australia | Product creation: adaptation of existing KMb product (Mandarin version of the Dementia and Driving Decision Aid booklet)  Literature/product search: NR  Stakeholder engagement: consultation was completed with experts as part of booklet development and translation; healthcare professionals, family caregivers and people with dementia completed pre and post-booklet surveys and experts participated in focus groups to provide feedback on the booklet  Resources utilized: experts reviewed booklets in terms of the content of translation, cultural appropriate modification and the booklet presentation  Evaluation: education programme was delivered to stakeholders who provided feedback on acceptability of booklet |
| Crouse  2023  Australia | Product creation: development of a new KMb product (APP for parents to promote cognitive and socioemotional development and well-being among child aged 0-5 years)  Literature/product search: thematic domains based broadly on another similar project; the cultural framework used summarized published literature (e.g., government reports, journal articles, and textbooks) that is relevant to child-rearing  Stakeholder engagement: workshops with in-country parents to further examine the acceptability, usability, and relevance of the content; iteratively revised based on feedback; the cultural framework and literature summary are reviewed and approved by an in-country partner organization  Resources utilized: nominated country-specific expert, parents and partner organizations; international experts in ethnography, anthropology, early childhood development, medicine, psychology, and other disciplines  Evaluation: each country conducted an evaluation via surveys examining impacts of APP on parent-level confidence and self-efficacy |
| Cunningham-Erves  2022  USA | Product creation: development of a new KMb product (library of messages targeting concerns around COVID-19 vaccines among African Americans)  Literature/product search: literature search to identify reasons for COVID-19 vaccine hesitancy and acceptance, along with potential strategies to improve coverage among AA  Stakeholder engagement: community involvement through all 6 phases; community-academic partnership between 2 academic partners, messaging pretesting with African Americas via semi-structured interviews  Resources utilized: interdisciplinary team of experts in basic science, epidemiology, behavioral science, communication, and community engagement; community advisory panel; physician; experts to review content  Evaluation: qualitative and quantitative methods in a 2-phase content review process by experts to evaluate relevance |
| Drago  2018  USA | Product creation: development of a new KMb product (decision aid for Latino parents facing imminent extreme premature delivery)  Literature/product search: literature review of published, relevant data on unique cultural aspects of Latino health care and communication barriers with Spanish-speaking patients created a theoretical framework to evaluate our findings  Stakeholder engagement: interviews with Latino parents of infants born before 26 weeks gestational age, with trained medical interpreter; interview themes and items were compared to those found in the development of the English decision-aid to identify novel themes to be addressed by the Spanish decision-aid; research team reviewed themes and adapted decision-aid through iterative process; certified medical transcription service was used to ensure accuracy of the Spanish wording on the adapted decision-aid, which was tested for face validity by four Spanish-speakers not participating in the study  Resources utilized: trained medical interpreter; certified medical transcription service  Evaluation: usefulness of Spanish decision aid assessed through simulated antenatal counseling session from the vantage point of a parent expecting the imminent delivery of an infant at 23-week gestation; member of the research team conducted the simulated counseling sessions in Spanish using a memorized script |
| Drenkard  2022  USA | Product creation: development of a new KMb product (Internet-based program to educate Latin American people about lupus)  Literature/product search: NR  Stakeholder engagement: rheumatologists from the Latin-American Group for the Study of Lupus collaborated with the selection of topics, community management, chats with experts, and dissemination; ILAR and PANLAR (the International and the Pan American League of Associations of Rheumatology) scientific organizations contributed to program dissemination; patients and patients' organizations participated in selection of topics, review of content, dissemination; community management team of junior rheumatologists participated in educational interactions with social media users, formative evaluation; production team  Resources utilized: rheumatologists; social communicator with expertise in social media; copywriter; community manager  Evaluation: through Facebook survey questions and traffic analysis |
| Du Plessis  2022  South Africa | Product creation: development of new KMb tool (visual illustrations of 12 food based dietary guideline themes among consumers in Tanzania)  Literature/product search: proposed food based dietary guidelines based on relevant scientific literature on the topic as well as country-specific literature for Tanzania  Stakeholder engagement: Tanzanian food based dietary guideline Technical Working Group comprising of academics and government officials from various sectors, United Nations, and non-governmental  organizations from the Republic of Tanzania developed 12 themes; the working group and researchers workshopped themes to come up with messaging; pilot field-testing was conducted as part of the fieldworker training; Tanzanian women from diverse backgrounds participated in focus groups to evaluate messages and images  Resources utilized: graphic designer; translators; academics and government officials  Evaluation: NR |
| Elliott  2022  Canada | Product creation: adaptation of an existing KMb product (digital whiteboard animation video depicting the signs and symptoms of croup for French and Filipino parents)  Literature/product search: NR  Stakeholder engagement: continual consultation with Filipino parent stakeholders  Resources utilized: graphic designers and key stakeholders (Filipino parents), translators  Evaluation: parents completed a usability survey and interview after viewing the adapted KMb tool to evaluate the video’s quality of information, format, appropriateness of visuals, and communication of health information |
| Glennie  2022  Australia | Product creation: development of a new KMb product (videos submitted by public about COVID awareness in remote Aboriginal communities of the Northern Territory of Australia)  Literature/product search: NR  Stakeholder engagement: Aboriginal and Torres Strait Islander Australians in specific areas of Australia where languages in videos were prominent  Resources utilized: local music production company; local advertising firm to distribute the videos via Facebook paid advertising  Evaluation: no formal evaluation but presented Facebook metrics and engagement levels |
| Gordon  2015  USA | Product creation: development of a new KMb product (culturally targeted website for Hispanic and Latinos)  Literature/product search: inspired by educational sessions targeted to Hispanic transplant patients that are provided by Northwestern University’s Hispanic Kidney Transplant Program; used theoretical approaches to guide website design  Stakeholder engagement: nine focus groups over three months with 76 Hispanic kidney transplant candidates living donors, dialysis patients, and members of the general public  Resources utilized: Northwestern faculty, including a medical anthropologist/ethicist with expertise in ethical issues relating to kidney transplantation and donation, health services researcher, instructional design health educators, a Hispanic transplant surgeon, and a Hispanic research staff member, and the National Kidney Foundation of Illinois, including the former Chief Executive Officer, the Hispanic community outreach staff member, and the former marketing expert; two additional staff members later assisted in translation processes, for a total of five Hispanic, bilingual team members  Evaluation: Usability testing via surveys was conducted by a third-party contractor with 18 Hispanic kidney transplant recipients and living kidney donors to assess users' ability to effectively navigate the website and satisfaction with the website design |
| Grasaas  2019  Norway | Product creation: adaptation of an existing KMb product (self-management APP;10-step process of preparation and forward translation to Norwegian and adaption of software interface text from Canadian English)  Literature/product search: NR  Stakeholder engagement: expert panel of researchers within field of pain ensured that two versions were conceptually equivalent, two adolescents assessed pain education library to ensure content was clear and easy to understand, review by end users after the usability field test to check its understandability and cultural relevance, review of cognitive debriefing, proofreading, and final report assessed by the project group  Resources utilized: experts in field of pain; translator  Evaluation: laboratory usability assessment: each participant completed 10 predefined tasks with the APP; completed System Usability Scale questionnaire; and interview guided by 14 open ended questions. Field usability test: five adolescents with persistent pain tested the APP continuously over a period of 2 weeks to assess user experience over time and to identify any need for further assistance while using the APP; answered electronic survey |
| Grinker  2015  USA | Product creation: adaption of an existing KMb product (Autism Speaks First 100 Days Kit for the Korean community)  Literature/product search: NR  Stakeholder engagement: unstructured individual and group interviews with Korean child health and education professionals to identify barriers to diagnosis and care, generate broad ideas about the range of cultural notions about developmental disorders; Korean mothers employed a version of “cultural modeling” or “culture as consensus” method, which assesses the degree to which individuals in a group share a set of beliefs; free- listening with Korean mothers  Resources utilized: child health and education professionals (psychologists, pediatricians, teachers, and social workers)  Evaluation: NR |
| Guttman  2013  Israel | Product creation: development of a new KMb product (health rights information materials for Ethiopian immigrants in Israel)  Literature/product search: NR  Stakeholder engagement: steering committee consisting of members from five advocacy organizations, four specifically working with the Ethiopian immigrant population themselves, Ethiopian immigrants, and a health rights organization; semi-structured group and personal interviews with community members; included four discussion sessions with steering committee members  Resources utilized: nurses, social workers, and health care liaison workers  Evaluation: NR |
| Hainsworth  2022  UK | Product creation: development of a new KMb product (recorded video for prostate cancer risk and screening for African or African-Caribbean ancestry)  Literature/product search: NR  Stakeholder engagement: discussion panel for development included three men who had participated in the screening study and four men who were members of the Black, Asian and Minority Ethnic staff forums; five participants from the screening study were filmed for the video  Resources utilized: participants who had participated previously in the screening study  Evaluation: impact of the video on recruitment of African and African-Caribbean men into the prostate cancer screening study |
| Hall  2022  Netherlands | Product creation: creation of a new KMb product (comic strips about diabetic retinopathy screening for those living in Kilimanjaro, Tanzania)  Literature/product search: prior needs assessment conducted with diabetics and health care workers in region to identify behavioural and environmental determinants of people with diabetes, health outcomes and quality of life  Stakeholder engagement: people with diabetes and community stakeholders were asked what format information for screening should take; members of the Kilimanjaro Diabetic Programme working committee developed scenarios for two comic strips and received feedback from the group; revisions of artwork were made with respect to culture and religion  Resources utilized: government representatives, endocrinologists, ophthalmologists, epidemiologists, social scientists, hospital directors, urban and rural health workers, and representatives of diabetics  Evaluation: level of comprehension and readability of the comic strips was tested by Flesch-Kincaid; use of the comic strips was piloted at Kilimanjaro Christian Medical Centre Hospital during a clinical trial for screening |
| Hamdiui  2021  Netherlands | Product creation: development of a new KMb product (three recorded videos on cervical cancer decision making among Turkish- and Moroccan Dutch women translated to Turkish, Moroccan-Arabic, and -Berber)  Literature/product search: NR  Stakeholder engagement: Turkish- and Moroccan-Dutch women participated in focus groups to develop the main themes, questionnaires were given to Turkish- and Moroccan-Dutch women to identify most relevant themes; Turkish- and Moroccan-Dutch women were shown unedited footage to verify whether the content and presentation matched their needs and requirements  Resources utilized: experts on cervical cancer; health communication experts; public health experts; Turkish and Moroccan languages/culture experts; video producer and director; semi‐professional actresses  Evaluation: RCT where participants were either given the normal brochure or the brochure and the new video. Participants completed an online questionnaire where they were asked questions about their informed decision-making and their opinions of the video |
| Harvey  2011  USA | Product creation: development of a new KMb product (Spanish written health brochures on common health information to promote health literacy)  Literature/product search: identified appropriate topics through Internet-based review of health information sources (public health authorities, federal agencies, disease-specific foundations) for model examples to inform development; systematic observation of patients at clinic; identified appropriate topics  Stakeholder engagement: interviews with staff and clinic patients about their experiences with written health materials; multiple revisions over the course of three months to ensure all content maintained high standards of medical accuracy and cultural and linguistic competence; convenience sample of 9 patients were asked their opinion on draft versions about ease of understanding, was content visually appealing, were images of families powerful  Resources utilized: volunteer graphic designer; Spanish speaking nurse; Physicians  Evaluation: brochures were evaluated using the Fernandez–Huerta readability equation |
| Hashim  2013  UAE | Product creation: development of a new KMb product (Arabic printed health education leaflets)  Literature/product search: NR  Stakeholder engagement: interviewer-administered questionnaire with Arabic-speaking participants recruited from clinic waiting areas, to measure design preferences (print format, fond, numerals and images); focus groups conducted with university administration, city zoo staff, and residents of local community  Resources utilized: experienced moderator  Evaluation: NR |
| Hempler  2015  Denmark | Product creation: development of a new KMb product (culturally sensitive dialog for diabetes management among Pakistani immigrants in Denmark)  Literature/product search: observation of individual education session; observing patient journey in the clinic  Stakeholder engagement: interactive workshop with dietitians and researchers; workshop for idea generation with researchers, an industrial designer, and dietitians; interviews with two patients and two health care professionals with Pakistani background; prototype feedback through interviews with patients and dietitians; testing of selected products in group-based patient education sessions; workshops; testing in individual patient education; final workshop  Resources utilized: public health researchers, behavioral and educational science; four dietitians, an industrial designer, a nutritional scientist with managerial responsibility  Evaluation: NR |
| Hodge  2012  USA | Product creation: development of a new KMb product (cancer symptom management toolkit for Southwest American Indians consisting of self-management guide, resource directory, and motivational video)  Literature/product search: NR  Stakeholder engagement: American Indian cancer survivors, family members, and others (caregivers, community leaders, and friends) participated in 13 focus group discussions; successive focus group built on the input of previous by reviewing preexisting cancer education materials and drafts of toolkit (videos, self-help guide, resource directory); participants were also asked to validate content, cultural acceptability, and presentation of artwork; American Indian communities in the Southwest videotaped first person narratives to develop video component and pilot tested by a focus group for review and feedback  Resources utilized: translators  Evaluation: forms to evaluate satisfaction, usability, and the cultural competency of the toolkit was disseminated among the participants who attended an educational talking circles intervention following development of the toolkit |
| Hong  2022  USA | Product creation: development of a new KMb tool (mHealth WeChat based intervention for Chinese American family caregivers of persons with dementia)  Literature/product search: NR  Stakeholder engagement: eight stakeholders including Chinese American family caregiver of persons with dementia, health care providers, and community leaders were engaged through weekly meetings to seek input about cultural appropriateness, ease of use, user engagement, and error reduction; tested and refined the product with five Chinese American family caregiver participants through an interview and ‘think out loud’ process.  Resources utilized: health care providers; community leaders; software engineer  Evaluation: NR |
| Jameel  2023  Australia | Product creation: development of a new KMb product (comic strips for pre-ED care to support Indigenous patients in a metro-urban ED in Melbourne, Australia)  Literature/product search: literature review conducted for understanding of the cultural safety needs of First Nations patients in ED environments; literature review exploring the types and usage of available illustration-based tools  Stakeholder engagement: First Nations project governance group that was consulted for the content areas; three online workshops were held to obtain feedback on the drafts from Indigenous community members.  Resources utilized: ED staff; professional illustrator  Evaluation: NR |
| Jiang  2021  USA | Product creation: adaptation of an existing KMb product (SMS text messaging for smoking cessation for Vietnamese smokers)  Literature/product search: NR  Stakeholder engagement: phase 1 included focus groups of Vietnamese smokers used to provide data on culturally relevant patterns of tobacco use and assess message preferences; phase 2 included a single-arm pilot test where participants received automated bidirectional text messages for 6 weeks and a random sample of participants were interviewed to inform additional changes  Resources utilized: community health collaborators from a health center in Hanoi  Evaluation: feasibility, engagement, and acceptability measured through surveys; interviews with participants who had received the text messages where they provided feedback for the program |
| Jiang  2021  USA | Product creation: development of a new KMb product (17 text messages delivered through WeChat for Chinese Immigrant smokers)  Literature/product search: NR  Stakeholder engagement: Chinese immigrant smokers participated in semi-structured in-depth interviews where research staff sent them text messages to read and rate on a scale of 0 to 10 to indicate to what extent the message enhanced their motivation to quit, promoted their confidence in quitting, and increased their awareness of quitting strategies; asked Chinese immigrant smokers for feedback on the text messages  Resources utilized: USA Clinical Practice Guidelines for Treating Tobacco Use and Dependence  Evaluation: NR |
| Kandasamy  2022  Canada | Product creation: development of a new KMb product (culturally sensitive videos on COVID-19 vaccines for South Asian youth in the greater Toronto and Hamilton area of Ontario, Canada)  Literature/product search: NR  Stakeholder engagement: a group of youth ambassadors comprised of young South Asian people (18-29) from Ontario communities created to evaluate the videos; received feedback from local grass-roots South Asian community organizations during development  Resources utilized: South Asian health researchers; epidemiologists; medical students; marketing specialists; video production team; community members were recorded for audio for the video  Evaluation: youth ambassadors were given a presurvey and a postsurvey to evaluate how the video had impacted their perceived knowledge on COVID-19 and their confidence in facilitating conversations with family and community members about COVID-19 vaccines and convincing vaccine uptake in their community |
| Kayler  2023  USA | Product creation: development and further refinement of a new KMb product (animated video series about kidney transplant and donation, KidneyTIME suitable for and acceptable to kidney transplant candidates and their support networks)  Literature/product search: review of published qualitative literature and online resources  Stakeholder engagement: during development scripts were revised based on input from local transplant providers; animations refined through cognitive interviews with transplant candidates/recipients, donors and their friends or family to determine suitability, with one component being cultural appropriateness; AA and non–AA patients were interviewed separately to identify components of the program and promote range in perspective; diverse individuals representing different ages, races, and ethnicities gave feedback on character illustrations; during refinement open-ended conversations were conducted with academic-community partners to gain input and feedback about how to refine video curriculum to ensure content was culturally sensitive, appealing, understandable, and met information needs; external stakeholders viewed animations and gave feedback, this feedback was discussed with steering committee and further revisions were made; experimental survey-pilot study of animation prototypes and questionnaire instruments was conducted among a convenience sample of 112 kidney transplant candidates  Resources utilized: *Development*: stakeholders (dialysis and transplant providers; community kidney advocates; kidney transplant recipients; donors); animator and illustrator; professional and non-professional voice actors, with some from within local community; community advisors that teach cultural competency; community transplant advocate; anthropologist; dialysis facility staff; *Refinement*: steering committee (community members consisting of those with kidney failure, living kidney donors, caregiver, dialysis social worker, local organ donation educator); purposeful sample of external stakeholders (transplant candidates, recipients, and their caregivers; dialysis staff; transplant providers; nephrologist); professional Spanish translator  Evaluation: experimental survey-pilot study of animation prototypes and questionnaire instruments was conducted among a convenience sample of 112 kidney transplant candidates |
| Kerr  2021  USA | Product creation: development of a new KMb product (multifaceted PrEP-focused HIV prevention messaging for AA young adults)  Literature/product search: NR  Stakeholder engagement: focus groups were conducted with AA who self-identify as belonging to the following groups: men who have sex with men, transgender women, high-risk heterosexuals (sex workers, people with HIV+ partners, non-users of condoms, persons with a history of sexually transmitted infection, persons who have a history of sexual concurrency or having partner engaged in sexual concurrency), and individuals from community-based organizations to gather thoughts about PrEP; a community advisory board helped guide the development of the intervention, they met with the research team for group discussion and provided preferences for campaign approaches, campaign verbiage and guided campaign esthetics based on ideas presented by the research team; a creative team composed of individuals from a corporate creative agency worked under the direction of research team members to develop options for campaign approaches, imagery, digital media and content, and Internet radio ads, a consulting firm composed of young AA from the local community also help develop social media content  Resources utilized: professional transcription service; marketing firm; community advisory board helped guide the development of the campaign  Evaluation: NR |
| Ko  2014  USA | Product creation: adaptation of an existing KMb product (colorectal cancer multimedia decision aid for Spanish speaking Latinos)  Literature/product search: review of relevant literature  Stakeholder engagement: group and individual meeting with regional stakeholders (researchers, gastroenterologists, promotoras, leaders/staff at regional community health centers); focus group with target population to adapt decision aid for a Spanish speaking audience; patient focus groups to solicit participant feedback and input about how the content, format, graphics, and individuals portrayed in the video could be adapted to increase relevance and message effectiveness  Resources utilized: video producer and actor  Evaluation: NR |
| LaMonica  2022  Australia | Product creation: development of new KMb product (Thrive by Five APP developed for parents and caregivers in 30 countries around the globe)  Literature/product search: comprehensive literature review about the cultures, traditions, and values of the people and the history and social context of the target country  Stakeholder engagement: University of Sydney partnered with Minderoo Foundation to support development of content; Minderoo identified an in-country partner to provide expertise throughout the project, including: guidance, information on local context, locating beta, assisting with APP implementation and promotion; in-country partners are expected to be a mix of governmental and nongovernmental organizations that align with the goals of Thrive by Five; in-country partners identified a subject matter expert group to provide feedback regarding the relevance and appropriateness for the local context  Resources utilized: Australian software development company; subject matter expert group (specialists in early childhood development and education, psychology, medicine, anthropology and representatives from relevant government ministries); translators  Evaluation: beta testing was done with a sample of parents, family members and key local stakeholders (e.g. preprimary schoolteachers and child psychologists) and they were asked to complete a questionnaire about their experiences using the APP; workshops were conducted to explore parent, caregiver feedback on the APP user experience, features, functions, and explore how the content can be developed for and tailored to the needs of the parents and caregivers in each country |
| Lee  2019  USA | Product creation: development of a new KMb product (mScreening APP among young Korean American immigrant women)  Literature/product search: NR  Stakeholder engagement: series of focus groups with young Korean American immigrant women to identify barriers and develop motivators and trigger for Pap test update; regular meetings with community advisory board members and mobile technology developers to get feedback and refine the mScreening APP  Resources utilized: community advisory board members (Korean American immigrant women in their 20s, a Korean American physician, a Korean American nurse, and religious and community leaders); bilingual Korean research assistant; mobile technology developers; mobile phone technology experts  Evaluation: NR |
| Leiter  2023  USA | Product creation: adaptation of an existing KMb product (multimedia educational intervention consisting of five videos and booklets for Spanish and English-speaking Latinos)  Literature/product search: conducted a systematic integrative review on psychosocial, cultural, and communication factors influencing illness understanding and treatment decision-making among Latino cancer patients  Stakeholder engagement: project was co-led by an oncologist/health services researcher and a Latina public health researcher focused on Latino health; interviewed Latino patients and their caregivers to obtain feedback  Resources utilized: multidisciplinary team of investigators, clinicians, and students; researchers with expertise; external advisors with expertise caring for Latino cancer patients; cross-cultural media production company; a firm with expertise in web-based behavioral health interventions  Evaluation: semi-structured interviewes with Latino patients with advanced cancer and their caregivers to solicit their feedback on the adapted multimedia |
| LeLaurin  2022  USA | Product creation: development of a new KMb product (RESCUE website to be used by those providing care for stroke patients in English and Spanish language)  Literature/product search: critically evaluated available stroke caregiver information in the literature, the Veteran Affairs patient portal, and other stroke and caregiver websites  Stakeholder engagement: materials were iteratively reviewed by experts in the content area, including physicians specializing in geriatrics, nurses, occupational therapists, a speech pathologist, a rehabilitation scientist, social workers, and a palliative care physician  Resources utilized: multidisciplinary team consisting of members with backgrounds in nursing, counseling, aging, and public health; physicians, nurses, speech pathologist, rehabilitation experts, social workers, translators; key leaders and partners within the Veterans Affairs, including National Center for Health Promotion and Disease Prevention, My HealtheVet National Stakeholders Manager, VA Care Coordination Office, Clinical Information Systems Specialist and Patient Education Lead, and a local Medical and Patient  Health Librarian, and outside Veterans Affairs the American Heart Association and American Stroke Association  Evaluation: usability testing with caregivers and healthcare providers; users were asked to review based on ease of use, locating topics, general navigation, clarity, attractiveness, readability, and content of the website |
| Lemon  2022  Australia | Product creation: development of a new KMb product (television commercials and radio advertisements in central Australia)  Literature/product search: NR  Stakeholder engagement: Aboriginal Community Controlled Health Service health promotion team developed scripts; Aboriginal community members in Alice Springs piloted the scripts using yarning sessions  Resources utilized: Aboriginal Community Controlled Health Service premises were used for filming; local community members were contracted as talent  Evaluation: NR |
| Li  2012  USA | Product creation: development of new KMb product (hypertension CD-ROM including visual aids and audio features for Chinese immigrants)  Literature/product search: conducted a literature review; accessed previous study findings; prior experience in hypertension management in older Chinese immigrants  Stakeholder engagement: first draft presented to healthcare providers and interdisciplinary health research team; materials revised based on experts’ suggestions  Resources utilized: interdisciplinary health research team (geriatric nurse scientist, gerontologist, epidemiologist, psychologist)  Evaluation: semi-structured individual and focus group interviews with Chinese immigrants conducted to evaluate the use of the product |
| Liu  2021  China/Canada | Product creation: adaptation of existing KMb product (Cardiac College^TM^ educational materials for Mandarin-speaking people living with cardiovascular disease in China and internationally)  Literature/product search: literature reviews on several topics, including a rapid grey literature search regarding best practices in translation and cultural adaptation; systematic review  Stakeholder engagement: group of healthcare providers (physician and nurses) reviewed the two translated versions, with the aim of identifying differences and similarities with the original version and ensuring cultural relevance and appropriateness, and made suggestions for change  Resources utilized: multi-disciplinary stakeholder team (patient education, translation and adaptation of materials, Cardiac College^TM^ developers, physicians with expertise in cardiac rehabilitation, Chinese cardiac care registered nurses, nurse experts, and a global leader in delivery of cardiac rehabilitation in low-resource settings); information specialists; translators (certified and bilingual healthcare professionals)  Evaluation: expert review of booklets using Patient Education Materials Assessment Tool for Printable Materials (PEMAT-P); patient review using think-aloud activity |
| Maertens  2017  USA | Product creation: adaptation of existing KMb product (*Teen VaxScene* culturally adapted for Latinx parents and youth; CHiCOS educational website)  Literature/product search: NR  Stakeholder engagement: community advisory committee modified Teen VaxScene, developed materials for focus groups and helped make iterative revisions to logo, color scheme and content; assisted in interpreting results from focus groups; and participated in analysis by clarifying meaning and completeness of group feedback; reviewed printed versions  Resources utilized: Latina parents of female adolescents and young adult Latinas  Evaluation: RCT to assess effectiveness of an online intervention to increase HPV vaccination intentions among patients of clinics that principally serve a Latinx population (Reno 2023) |
| Malamsha  2021  United Republic of Tanzania | Product creation: development of a new KMb product (HappyToto mobile-based game for educating young children [< 5 years], parents and caretakers on sexual abuse prevention)  Literature/product search: NR  Stakeholder engagement: Tanzanian parents were given questionnaires to contribute to the design of the game user-interface; parents and children gave feedback about the game; child care experts were included in focus groups to help develop the game content  Resources utilized: Dart programming language; Adobe After Effect; Adobe Illustrator  Evaluation: Tanzanian parents and children participated in surveys, interviews, and observations while using the game to validate the game by examining acceptability, pre- and post-confidence and ability scores, and usability |
| Martinez  2023  USA | Product creation: development of a new KMb product (pamphlets on behavior interventions for parents of children with ASD)  Literature/product search: a review of the literature regarding behavior analytic interventions, cultural adaptation of health interventions and materials, and comprehensibility of health documents  Stakeholder engagement: 4 mothers of autistic children were given a survey to assess their preferences on the materials; 2 mothers of autistic children were assessed to study the effects of the materials on their performance during rehearsal of behavioral techniques  Resources utilized: three subject matter experts composed of PhD level practitioners and professors reviewed the materials and provided feedback  Evaluation: mothers were given the pamphlets and their performance of the behavioral techniques was measured to see how effective the materials were at teaching the techniques |
| Materia  2020  USA | Product creation: development of a new KMb product (SMS text messages to encourage healthy behaviors for diabetes prevention)  Literature/product search: literature searches were conducted to investigate types of messages to use and testing practices  Stakeholder engagement: an advisory board was made up of multiple stakeholders including local residents, community health workers, and local leaders from the Khayelitsha community which were advised throughout the entire resource development  Resources utilized: South African community advisory board; professional translators; technology developer  Evaluation: NR |
| Mathieson  2012  New Zealand | Product creation: adaptation of an existing KMb product (cognitive behavioural therapy self-management for Maori)  Literature/product search: review of the literature  Stakeholder engagement: partnership was formed with a Maori health researcher; researchers established relationships with local providers, conducted interviews with patients and clinicians, and had face-to-face contact with patients to collect intake and outcome data; semi-structured interviews conducted with nine general practitioners and nine primary care nurses to review existing material and suggest how it would need to be adapted to better meet needs of Maori patients; semi-structured, individual face-to-face interviews conducted with six potential patient users  Resources utilized: Maori health researcher; graphic designer  Evaluation: clinician and patient satisfaction questionnaires, based on a five-point Likert scale to indicate levels of agreement or disagreement; both patient and clinician feedback had suggested that full translation would not be helpful |
| Mauka  2021  Tanzania | Product creation: development of a new KMb product (mHealth APP on PrEP adherence among East Africa)  Literature/product search: literature review for apps to address adherence of PrEP; literature review on mobile phone access and usage involving the target population (African)  Stakeholder engagement: members of the target population were consulted for problem definition and justification of the app’s relevance; end-user group discussions for logo design; pilot testing focus groups with target population to explore perceptions, experiences, and challenges associated with the app (rigor cycle);  Resources utilized: team of experts in HIV behavior and research reviewed the app; prototype evaluators including PhD students, medical researchers, epidemiologists, biostatisticians, public health specialists, and clinical research coordinators.  Evaluation: usability was measured through user statistics of various APP features |
| McFarlane  2019  USA | Product creation: development of a new KMb product (CChiRP website, four animations, and a decision aid to assist decision making for Hispanic cancer patients)  Literature/product search: NR  Stakeholder engagement: Hispanic cancer patients were shown the website, videos, and decision aids and given the chance to provide feedback  Resources utilized: prototype resources developed by an established health communication scholar; an expert in interactive media production, a health economist specializing in decision-making, a computer scientist, an oncologist experienced with issues of clinical trial accrual, an expert in organizational change, an industrial engineer who specializes in human factors, a statistics expert, and a representative from the cancer support community  Evaluation: focus groups were conducted with Hispanic cancer patients to receive their feedback and recommendations about the KMb materials |
| Meherali  2021  Canada | Product creation: development of a new KMb product (whiteboard animation video on AOM for Pakistani parents)  Literature/product search: systematic review to determine the information needs of parents whose children have acute otitis media (AOM)  Stakeholder engagement: 16 individual qualitative interviews with Pakistani parents who sought care for AOM in a hospital ED to understand their information needs  Resources utilized: storywriter, graphic designer, editor, translator  Evaluation: mixed-methods evaluation process (surveys and focus groups) to determine the usability, usefulness, and cultural appropriateness of the KMb product for Pakistani parents |
| Montague Lecturer  2022  UK | Product creation: development of a new KMb product (1-minute mobile digital animated video)  Literature/product search: NR  Stakeholder engagement: asylum seekers were interviewed to evaluate the animation for social and cultural sensitivity and ability to increase intention to change behaviours in order to increase physical activity  Resources utilized: peer interpreters, volunteer from a community organization which supports asylum seekers  Evaluation: asylum seekers were interviewed to examine feasibility of the animation and provide feedback on social and cultural elements |
| Norris  2021  USA | Product creation: adaptation of existing KMb product (Mighty Girls APP to support sexual health behavior change)  Literature/product search: NR  Stakeholder engagement: consultants to review Mighty Girls with a female middle school staff member and adult women (two African American women and one biracial African American/Native American); girls enrolled in the 7^th^ grade at two multiethnic middle schools participated in implementation  Resources utilized: research assistants were female middle school staff members and three adult women in their early 20s (two AA women and one biracial AA/Native American woman)  Evaluation: usability testing (adapted System Usability Scale) performed at each school using their own Android phone or one provided by research team; focus groups with parents to gauge support |
| Pathak  2021  USA | Product creation: development of a new KMb product (text messages for use within an adaptive smartphone APP for low-income ethnic minority patients about diabetes)  Literature/product search: scanned literature on crowdsourcing to inform development of text messaging for low income minorities  Stakeholder engagement: five phase iterative design process: (1) feasibility tested by English-speaking and Spanish-speaking primary care adult patients (with diabetes and depression) recruited from a safety-net health care setting; (2) technology acceptance pilot testing with English-speaking and Spanish-speaking patients with comorbid diabetes and depression recruited from the same primary care setting as phase 1; (3) crowdsourcing used to test for motivational construct categorization; (4) expert evaluation by the research team  Resources utilized: NR  Evaluation: pilot testing done by patients and via crowdsourcing to complete a survey |
| Payan  2020  USA | Product creation: development of a new KMb product (Spanish educational brochure on breast cancer information for patients, CHW-delivered intervention)  Literature/product search: identified literature and evidence-based guidelines to inform selection of the channel, receiver, and destination  Stakeholder engagement: a community advisory panel consisting of local and national organizations with expertise in health communication and/or health education materials for Latinos, patients, and medical experts provided feedback on the prototype brochure  Resources utilized: bilingual and bicultural Latina research staff and medical experts  Evaluation: participants breast cancer risk knowledge, perceived breast cancer susceptibility, and self-efficacy to access breast cancer-related advice or information were assessed and compared to a control group |
| Povey  2022  Australia | Product creation: enhancement of previously developed KMb by same research group (e-MH tool)  Literature/product search: narrative literature synthesis was undertaken to inform APP development  Stakeholder engagement: young people and service providers recruited from 2 schools and 1 residential drug rehabilitation facility; separate group of young people to form an Indigenous youth reference group; Indigenous researchers; existing expert reference group consisting of service providers and researchers with relevant expertise from MH, drug and alcohol, child protection, and education sectors; workshops assessed paper-based 2D version of APP  Resources utilized: Indigenous interpreter and APP developers  Evaluation: A current feasibility study is underway to evaluate the usability, feasibility, and appropriateness of the APP and its potential for improving the well-being of Aboriginal and Torres Strait Islander young people. |
| Quintana  2022  Argentina | Product creation: development of a new KMb product (bilingual audiovisual material for patients with RA in Argentina)  Literature/product search: NR  Stakeholder engagement: community collaborated in the process of production; reached a consensus on video animations, music, colors, and general esthetics  Resources utilized: community representatives; audiovisual designer  Evaluation: survey and comments via Facebook and WhatsAPP implementation |
| Rami  2018  Egypt | Product creation: adaptation of an existing KMb product (schizophrenia leaflet for Egyptian patients)  Literature/product search: research team undertook a literature review of theoretical and intervention papers to identify important factors for successful cultural adaptation of psychosocial interventions in schizophrenia  Stakeholder engagement: adapted with patients and caregivers  Resources utilized: Egyptian patients meeting DSM-IV criteria for schizophrenia and at least one caregiver  Evaluation: pilot tested with 20 patients who were not included in the main study to examine acceptability and linguistic accessibility of the products and educational materials and further modifications were made to improve the fit with the Egyptian cultural context |
| Santos  2021  Brazil | Product creation: adaptation of an existing KMb product (booklet for children undergoing peripheral intravenous catheterization)  Literature/product search: scoping review that addressed the concept of the peripheral intravenous catheterization and strategies for coping with the child undergoing procedure  Stakeholder engagement: committee of expert judges (nurses and doctors) validated items related to motivation, culture and applicability  Resources utilized: NR  Evaluation: validation by expert judges |
| Sharpe  2013  USA | Product creation: development of a new KMb product (educational brochure for American Indian Women)  Literature/product search: literature review on women’s knowledge of human papillomavirus, pap tests, and cervical cancer  Stakeholder engagement: worked with the Cherokee Women’s Wellness Center (CWWC) staff to develop and evaluate educational material; identify communication strategy with audience; through in-depth interviews gathered audience specific input  Resources utilized: CWWC nurses, tribes’ graphic artist, rural, primary health care clinics  Evaluation: establish criteria for evaluation of existing materials based on review of educational materials; pretesting done with women to assess perceived meaning, format, appearance and comfort level with the content, and cultural relevance |
| Songtaweesin  2021  Thailand | Product creation: adaptation of existing KMb product (P3 [Prepared, Protected, empowered] APP)  Literature/product search: NR  Stakeholder engagement: young Thai men who sleep with men, and service providers gave feedback about P3 and offered suggestions for adapting it to create a new APP (P3); focus groups with young Thai men who have sex with men and interviews with key informants  Resources utilized: key informants included counselors, physicians, nurses and a social worker; Thai collaborator skilled in graphic design, non-governmental organization to produce videos  Evaluation: NR |
| Stanley  2018  USA | Product creation: adaptation of existing KMb product (paper based messaged reducing substance use for American Indian Youth)  Literature/product search: NR  Stakeholder engagement: campaign discussed with Indigenous community advisory committee who provided input on campaign development and implementation, focus groups with 7^th^ graders who ranked examples of text and visuals, participants were asked about best ways to deliver campaign messages to them and their use of technology; high school students given disposable cameras to take pictures corresponding to questions, photos discussed with staff  Resources utilized: Indigenous community members, school staff  Evaluation: NR |
| Teles  2021  Portugal | Product creation: adaptation of existing KMb product (iSchool online self-help programme for informal caregivers of people with dementia)  Literature/product search: conducted a review of empirical studies on the characteristics and psychosocial needs of informal caregivers of people with dementia  Stakeholder engagement: adaptation was conducted in partnership with the Portuguese national Alzheimer Association  Resources utilized: professional translator; national statistics of the Portuguese population  Evaluation: the translated and adapted version of iSupport was independently reviewed by a panel of four national experts on ageing, dementia and psychosocial and/or psychological support for informal caregivers of people with dementia |
| Tolentino  2022  USA | Product creation: development of new KMb products (videos, posts and infographics of COVID-19 relevant public health topics that were spread through social media)  Literature/product search: NR  Stakeholder engagement: Native Hawaiian, Pacific Islander and Filipino youth Public Health Ambassadors were engaged to serve as positive and trusted social media influencers to deliver personalized message; partners, ambassadors and community gave feedback on COVID-19 public health topics  Resources utilized: high school students and young adults (Public Health Ambassadors), partner organization, community members  Evaluation: NR |
| Umaefulam  2022  Canada | Product creation: adaptation of an existing KMb product (decision aids for early detection of RA)  Literature/product search: NR  Stakeholder engagement: A cohort of First Nations person with lived experience of RA were interviewed; a second cohort was recruited from rheumatology clinic located at an urban Indigenous primary healthcare center participated in interviews to affirm alignment with the changes made to the original decision aid and suggested further adaptations to be made  Resources utilized: research team consisting of female Indigenous health researchers; First Nations person with lived experience of RA, a Métis rheumatologist and health services researcher, and two rheumatologists  Evaluation: NR |
| Valenzuela-Araujo  2021  USA | Product creation: evaluation of an existing KMb product (nine-minute Spanish language educational video supporting healthcare navigation and engagement skills of Spanish-speaking Latino parents of infants)  Literature/product search: NR  Stakeholder engagement: Latino family advisory council met with design fellow to inform video design and content; topics discussed at sessions included desired video content, length, script, character appearances, background music, and cultural appropriateness; evaluation survey pilot-tested by advisory council  Resources utilized: design fellow from Maryland Institute College of Art; Latino family advisory council  Evaluation: parent evaluation of video style and content |
| van der Steen  2013  Canada | Product creation: adaptation of an existing KMb product (dementia booklet on palliative care issues for implementation in Italy, Netherlands and Japan)  Literature/product search: NR  Stakeholder engagement: local teams (researchers, ethicists and physicians) in dementia palliative care participated in the translation and subsequent adaptations in the final text in English for analyses  Resources utilized: professional translators, researchers, ethicists, physicians, nursing home staff  Evaluation: an international acceptability study was initiated, evaluating and comparing acceptability and usefulness of the adapted and original versions among families and practitioners |
| Van Son  2014  USA | Product creation: adaptation of an existing KMb product (twelve diabetes educational materials for older Russian-speaking immigrants, based on existing handouts such as food exchange lists, self-care, physical activity, and menu-planning guides)  Literature/product search: NR  Stakeholder engagement: Russian cultural consultants gave input on materials; focus group of Russian speaking immigrants evaluated the readability, word selection, illustrations, and cultural relevance of the materials; diabetes educators and clinicians in various clinic settings volunteered to use and evaluate the drafted materials  Resources utilized: Russian-speaking health care providers, a home health nurse, a certified diabetes nurse educator, Russian speaking interpreters, a certified diabetes dietitian educator, a community education expert, a nurse gerontologist, and a physical therapist, certified medical translators  Evaluation: focus groups and telephone survey used to evaluate materials, with male and female Russian-speaking immigrants older than 65 years with diabetes, older Slavic immigrants and providers |
| Versteegh  2022  Australia | Product creation: adaptation of an existing pictorial-based asthma flipchart KMb product (multilingual asthma APP in several local First Nations languages and English)  Literature/product search: NR  Stakeholder engagement: nurses and medical professionals mapped out asthma APP agreeing on framework, functionality, and content based on previous culture-specific pictorial-based asthma flipchart; First Nations Reference Group approved readability, accuracy and cultural appropriateness; First Nations health professionals and non-health professionals testing for usability, functionality, translation issues  Resources utilized: respiratory research nurses, First Nations nurse, medical professionals experienced working with First Nations people, Menzies School of Health Research Australian First Nations Reference Group for Child Health and First Nations Health professionals; Linguists, from the Northern Territory Aboriginal  Interpreter Service; APP developer  Evaluation: Australian First Nations health professionals participated in semi-structured interviews to evaluate whether the Asthma APP improved knowledge and understanding of asthma among First Nations carers of children with asthma; carers underwent a pre-education questionnaire on asthma knowledge, followed by education using the Asthma APP, and then a post-education questionnaire immediately after. |
| Wall  2022  USA | Product creation: development of a new KMb product (educational video tailored for Black men about organ donation)  Literature/product search: NR  Stakeholder engagement: community advisory board advised on recruitment and video production; production team casted and drafted story boards, with content prioritized to address beliefs and misconceptions identified  from the formative interviews  Resources utilized: community advisory board comprised of barbershop owners, activists, and clergy; professional health media company; educational-entertainment experts; PhD registered nurse; physician  Evaluation: RCT to assess whether generic versus culturally targeted or personally tailored educational videos,  produced for Black men in Black owned barbershops, differentially affect organ donor registration after viewing |
| Wright  2023  Canada | Product creation: development of a new KMb product (knowledge dissemination tools in the form of a video series to share Indigenous mothers’ important messages about their experiences with healthcare providers to healthcare providers)  Literature/product search: NR  Stakeholder engagement: Advisory board (First Nations and Inuit mothers, First Nations early childhood service providers, First Nations Knowledge Holder, health providers, First Nations and non-Indigenous researchers) met several times to determine the purpose of the video series, review and select video production team and create content maps for each video; two videos and trailer reviewed by Advisory board and provided final approval of video and website  Resources utilized: video production team (videographer production manager)  Evaluation: NR |
| Wu  2021  Australia | Product creation: development of a new KMb product (six A5-sized booklets for Chinese-Australian immigrant affected by cancer with various coping skills and strategies for healthcare communication, treatment decision, managing physical symptoms, emotions and relationships, and finding additional support)  Literature/product search: informed by systematic review findings of Chinese patients’ and caregivers’ unmet supportive care needs  Stakeholder engagement: feedback sought from community in two rounds using focus groups and semi-structured interviews; during the first consultation, participants provided feedback to inform revisions to the first two draft booklets and further development of the remaining booklets; during the second consultation, participants provided feedback on revised booklets and remaining drafts, resulting in the final iteration  Resources utilized: accredited translators and interpreters  Evaluation: NR |
| Yeager  2022  USA | Product creation: development of a new KMb product (two videos, one featuring Black and the other white breast cancer patients participating in clinical trials)  Literature/product search: NR  Stakeholder engagement: advisory board met throughout video development phase to decide on information sources, the content, and format  Resources utilized: community advisory board (mostly breast cancer patients that had participated in clinical trials); video cast consisted racially diverse cancer patients, oncologists, and research coordinators; two Black cancer patients were cast in second video  Evaluation: pre-post survey with 50 cancer patients asking about future participation in clinical trials and a short survey to evaluate the video |
| Zerafa  2022  Australia | Product creation: development of a new KMb product (animated videos, showcasing three types of incontinence: urgency, stress and frequency)  Literature/product search: NR  Stakeholder engagement: co-design participants via workshops generate ideas and build early-stage content prototypes; prototypes were refined and tested; final prototypes were further designed and developed into a series of videos  Resources utilized: cross-section of consumers to share their wisdom and experience to design the resource  Evaluation: NR |

Abbreviations: African American = AA; Application = APP; Breast Cancer Screening = BCS; Coronavirus = COVID; Emergency Department = ED; Human Immunodeficiency Virus = HIV; Human Papilloma Virus = HPV; Knowledge Mobilization = KMb; Knowledge Translation = KT; Mental Health = MH; Not Reported = NR; Pre-Exposure Prophylaxis = PrEP; Randomized Controlled Trial = RCT; Rheumatoid Arthritis = RA; Short Message Service = SMS; United Kingdom = UK; United States of America = USA
