# Supplementary material for: Exploring methods for creating or adapting knowledge mobilization products for culturally and linguistically diverse audiences: a scoping review
Source: Arch Public Health. 2024 Jul 22;82:111. doi: 10.1186/s13690-024-01334-0 (PMC11265177; doi:10.1186/s13690-024-01334-0)
Supplement: Supplementary file 6 — Supplementary Material 6. [file 13690_2024_1334_MOESM6_ESM.docx]

**Additional file 6: Researcher reflections on creation or adaptation process**

| **Study** | **Researcher Experiences** |
| --- | --- |
| Abascal-Miguel  2022  Guatemala | “Our partnership with Wuqu’ Kawoq enabled us to find sustainable solutions for a priority of the organisation and community.” A well- balanced partnership that is open and honest about power differentials, money, and time has the potential to create and sustain impact. By involving the community in every step of the research process, we could tailor the intervention to them and gain the participants’ trust. |
| Ali  2018  UK | “The use of a co-design approach was helpful in terms of putting in place linkages with community-based organizations and venues through which the resultant materials could be distributed” |
| Avila  2023  USA | “Challenges with regional variation Spanish translation.” |
| Cabassa  2012  USA | “Feedback from stakeholders kept our team grounded to make sure that the fotonovela was entertaining and acceptable to our audience”  “The incorporation and balancing of the opinions and feedback from different stakeholders was not an easy task” |
| Drenkard  2022  USA | Considerable time commitment by production and community management teams and multiple stakeholders to reach out and engage a broad audience; “we could not directly measure if our educational resources lead to increased knowledge or behavioral change”. |
| Du Plessis  2022  South Africa | “Challenges included incorporating different names and cooking methods and representing different clothing.” |
| Hainsworth  2022  UK | “Failure to engage men fully through methods used, women were more likely to take information and pass along to male family members.”  “Barber shop setting was a novel idea that challenged preconceptions and led researchers to venture outside of comfort zone of biomedical setting.” |
| Hall  2022  Netherlands | “The level of readability of the comic strips could have been higher. Comprehension tests of the comic strips were not undertaken with the target group of PWD due to limited time and resources.” |
| Hempler  2015  Denmark | “This process required a great deal of dialog between researchers and dieticians. Dieticians had a strong focus on the outcome, namely effective education, as opposed to the researchers’ strong focus on the process leading to effective education through designing, testing, and redesigning products.” |
| Hodge  2012  USA | Improving communications between American Indian cancer survivors and healthcare  providers, as well as with family and friends, was identified as a major focus for the toolkit. |
| Kandasamy  2022  Canada | “It was important to foster collaborations between academia (to understand  evidence) and marketing and communications (to craft and disseminate message).” |
| Kayler  2023  USA | “Regular meetings fostered relationships among the team and aided in clear communication and confidence about what needed to be included in the education.”  “Discussions were lively and engaged, however we faced contextual debate in making the animations. Some participants desired more extensive content, whereas others felt the information should focus on fundamental concepts.” |
| Kerr  2021  USA | “Challenge was the misalignment of community/campaign sensibility and corporate interest; with some language deemed potentially problematic by print media vendors.” |
| Leiter  2023  USA | “Process required a large team possessing significant linguistic, ethnic, professional, and disciplinary diversity. Which was made possible through resources and infrastructure with established partnerships.” |
| LeLaurin  2022  USA | “Establishing partnerships with key stakeholders was key to ensure materials were comprehensive, accessible, and appropriate for use in routine clinical care.”  “These mutually beneficial partnerships  were also instrumental in establishing the credibility of the website and providing strategies for promotion and sustainment.”  “Despite our best efforts to effectively translate the RESCUE materials, Spanish speaking caregivers and providers were able to identify opportunities to improve the Spanish-language adaptation of the website, demonstrating that simple translation is not sufficient.” |
| Lemon  2022  Australia | “Prioritising the comfort of participants was critical to obtaining feedback.” |
| Liu  2021  China/Canada | “In terms of best practices for cultural adaptation of educational materials in the same language but for use in different settings, our group recommends: (1) involve stakeholders throughout process; (2) learn as much about target population; (3) pilot materials with target population; (4) document entire process for transparency and potential future deliberation.” |
| Martinez  2023  USA | “Expanding materials to be inclusive of a larger variety of ethnicities would improve the external validity of findings.” |
| Materia  2020  USA | “Multiple stakeholders should be identified and engaged throughout system creation and testing.” |
| Mathieson  2012  New Zealand | “The experience of this adaptation was rewarding for the research team and offers a model for other areas of clinical practice.” |
| Montague Lecturer  2022  UK | “Iterative co-design could have avoided language and dialect errors, and the animation and messages might have been refined more appropriately from the start.” |
| Pathak  2021  USA | “It is valuable to leverage crowdsourcing platforms as a more accessible and inexpensive source of feedback.”  “We found that expert input and review rendered cohesiveness and reliability to the  design process” |
| Povey  2022  Australia | “Maintaining young people’s participation for 2 years during adolescence, where  social, emotional, and vocational needs are changing, was difficult.”  “The staggered timing of diverse groups across multiple sites and the iterative nature of participatory design challenged our democratic processes.”  “Budget and timeline restrictions limited what was incorporated into this first prototype app design.”  “Determining the degree to which upskilling of participants occurred in a culturally and linguistically diverse, iterative situation, where knowledge from all stakeholders remained both tacit and latent, presented a significant challenge.” |
| Quintana  2022  Argentina | “This study also shows some of the barriers shared by other authors for the implementation of virtual strategies as educational tools: verbal or non-verbal communication barriers; digital barriers; and above all the impossibility of sustaining  the relationship created with the community.” |
| Rami  2018  Egypt | “Positive perspectives of adapted information materials do not guarantee adherence to programs, particularly with culturally stigmatized conditions like schizophrenia.” |
| Songtaweesin  2021  Thailand | “Planning for a budget that is sufficient for formative research and app adaptations may ensure that the adapted app is culturally and contextually relevant and may shorten the time between adaptation, pilot study, and, if feasible and acceptable, a larger trial.”  “Including local designers and developers on the team may help stretch limited resources and ensure that local expertise leads to the cultural adaptation process.”  “Having a fluent Thai speaker on the creation team could help address these and similar barriers in the adaptation process.” |
| Teles  2021  Portugal | “In spite of the participation of key stakeholders, we consider that the involvement of  informal caregivers of people with dementia, the programme’s end-users, would be required to claim for a bottom-up approach.” |
| Tolentino  2022  USA | “Creating content and scheduling them across platforms takes time and expertise.”  “Visuals are important but time-consuming.”  “Social media reached a wider audience, broadening the reach and providing valuable information in a cost-effective way, but resulted in more anti-vaccine and health misinformation added in the comments of the boosted posts.”  -“Another key lesson was learning about timing and perspective. Many community members are on their own timelines of understanding and acting upon public health information and resources because of a history of mistrust and resentment towards the government and other decision-making entities.” |
| Valenzuela-Araujo  2021  USA | “Using previously established community relationships via a clinic advisory council provides an example model for ensuring a strong partnership with community members that results in a product that is relevant to the target population.” |
| Wright  2023  Canada | “Researchers must recognize the potential for retraumatization when engaging Indigenous research participants in disseminating research findings. “Although dissemination activity was conceptualized and created by Indigenous mothers, it was not pre-emptively realized that this would potentially be harmful.” |
| Wu  2021  Australia | “Cultural congruence is more effectively achieved by working with the target community. Focus groups and semi-structured interviews are useful strategies to engage with the community.”  “When designing self-management resources and other interventions for immigrant groups, the dyadic role of the caregiver and patient should be considered.” |
| Zerafa  2022  Australia | “Through the co-design process we learned that it was important to consider inclusion, storytelling, transparency, empowerment, and dignity.” |
